# Antifibrotic mechanism of curcumin and its therapeutic potential in multi-organ diseases

**DOI:** 10.3389/fphar.2026.1691999

**Published:** 2026-08-24

**Authors:** Ting Luo, Jia Gao, Qingzhi Liang, Yi Su, Xiaoqin Liu, Shu Ou, Yaowen Zhang, Hong Gao, Yi Fu, Chunguang Xie

**Affiliations:** 1 Hospital of Chengdu University of Traditional Chinese Medicine, Chengdu, Sichuan, China; 2 TCM Regulating Metabolic Diseases Key Laboratory of Sichuan Province, Chengdu, Sichuan, China; 3 The Third Affiliated Hospital of Yunnan University of Chinese Medicine, Kunming, Yunnan, China; 4 Department of Endocrinology, Hospital of Chengdu University of Traditional Chinese Medicine, Chengdu, Sichuan, China

**Keywords:** curcumin, fibrosis, natural products, pharmacological mechanism, treatment

## Abstract

Fibrosis is the outcome of chronic diseases and manifests as an abnormal repair process in which normal parenchyma is progressively replaced by deposited extracellular matrix. It leads to organ dysfunction and is associated with high morbidity, disability, and mortality, thereby becoming a major public health concern. Traditional Chinese medicine shows multi-target, multi-pathway strategies with favorable safety profiles. Curcumin (CUR), a polyphenolic metabolite derived from *Curcuma longa L.* (Zingiberaceae), has been shown to modulate key fibrotic signaling pathways, including AMPK, autophagy, extracellular-regulated protein kinase (ERK), transforming growth factor β (TGF-β)/Smad, JNK, and Wnt/β-catenin. However, a critical analysis of more than 200 included studies reveals that the current evidence base is fundamentally descriptive rather than conclusive. The data show reproducible antifibrotic signals in acute chemical injury models (bleomycin (BLM), carbon tetrachloride (CCl_4_), and streptozotocin (STZ) when CUR is administered prophylactically. The data do not show (1) efficacy in chronic, progressive disease models that recapitulate human pathology; (2) a dose–response relationship linking achievable tissue concentrations to antifibrotic effects; (3) superiority over or an add-on benefit to standard-of-care antifibrotics; or (4) robust clinical efficacy beyond small, uncontrolled case series. Despite this promising preclinical evidence supporting CUR’s antifibrotic efficacy, clinical translation remains constrained by several critical limitations. The majority of evidence derives from *in vitro* studies using supraphysiological concentrations that far exceed achievable human plasma levels and from animal models that incompletely recapitulate the chronic, progressive nature of human fibrotic diseases. Additionally, the inherently poor oral bioavailability of CUR—despite advances in formulation strategies—remains a persistent obstacle. A critical appraisal of the existing literature further reveals substantial heterogeneity in experimental designs, a predominance of positive results suggestive of publication bias, and insufficient mechanistic validation to establish causality. This review outlines the mechanisms of action, safety, adverse effects, drug interactions, and the application of CUR-related nanocomposite products, thereby providing a foundation for in-depth research on its antifibrotic properties and clinical application.

## 1 Introduction

### 1.1 Overview of fibrosis

Fibrosis is a pathological process characterized by inflammation injury with continuous stimulation and excessive tissue repair leading to fibrous connective tissue remodeling, which is characterized by aberrant fibroblast activation, abnormal proliferation, high expression of collagen fiber proteins, and abnormal deposition of the extracellular matrix (ECM) (Zhang et al., 2020; Zhang and Zhang, 2020). Fibrotic diseases can affect various organ systems, including the respiratory, digestive, circulatory, and urinary systems. With the progression of the disease, the excessive deposition of ECM in organs destroys or even replaces parenchymal tissues, forming permanent scars, resulting in organ dysfunction and ultimately organ failure (Raghu et al., 2022), thereby causing respiratory failure, liver cirrhosis, heart failure, renal failure, polycystic ovary syndrome (PCOS), and other chronic diseases (Weiskirchen and Tacke, 2019). Despite organ-specific pathophysiological niches, fibrosis across different organs converges on a limited set of core signaling cascades. The transforming growth factor beta (TGF-β)/Smad pathway serves as the central driver of myofibroblast activation and extracellular matrix deposition. The nuclear factor-like 2 (NRF2) antioxidant axis and the PPARγ-mediated metabolic–inflammatory network exert major modulatory effects, primarily by counteracting TGF-β signaling or mitigating oxidative stress. Additional pathways, including NF-κB, PI3K/AKT, and Wnt/β-catenin, are also implicated. However, the relative contribution and crosstalk of these pathways vary considerably by etiology and organ microenvironment, and integrated analyses of their interplay with autophagy or apoptosis remain scarce. Rather than reiterating these basic mechanisms in each organ-specific section, the following sections will focus on organ-specific deviations, pathway dominance shifts, and knowledge gaps unique to each organ. This thematic overview therefore provides a mechanistic baseline to avoid redundancy and enable comparative analysis across organs.

### 1.2 Curcumin

**FIGURE 1:**
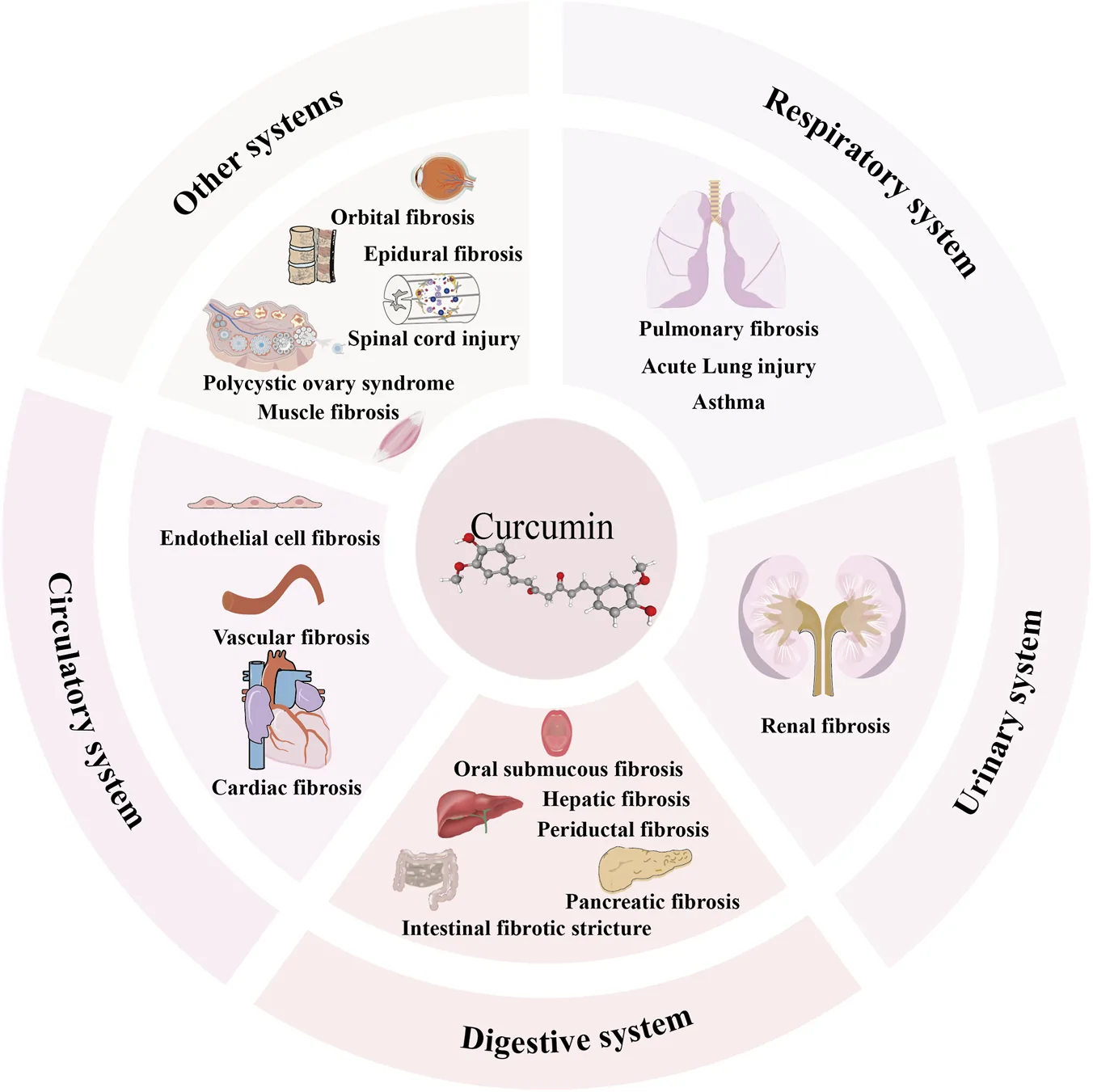
Chemical structure (3D) of curcumin (CUR). CUR is effective in multisystem organ fibrosis. All figures were drawn, arranged, and annotated using Adobe Illustrator 2020.

Turmeric is the rhizome of *Curcuma longa L.* belonging to the family Zingiberaceae. It has a firm texture, a golden yellow cross section, and a strong aroma and was first documented in Xinxiu Materia Medica (New Cultivation of the Materia Medica). In traditional Chinese medicine, it is considered hard and warm in nature, entering the spleen and liver meridians, with the effects of promoting qi circulation, relieving pain, breaking blood stasis, and promoting menstruation. Curcumin (CUR) is a polyphenol metabolite extracted from the rhizomes of *Curcuma longa L.*, *C*. *wenyujin*, *C*. *guangxiensis*, and *C*. *phaeocaulis* (family Zingiberaceae), with a content ranging from approximately 2% to 8%. As a natural polyphenol, CUR possesses intrinsic anti-inflammatory, antioxidant, and anti-apoptotic properties. Through coordinated modulation of multiple signaling pathways [e.g., AMPK, NRF2/heme oxygenase 1 (HO-1), NF-κB, and TGF-β/Smad], CUR can simultaneously suppress myofibroblast activation, reduce ECM deposition, and promote the resolution of established fibrosis. These mechanistic advantages position CUR as a promising multi-organ antifibrotic candidate, offering potential benefits over single-target therapies, particularly in complex, progressive fibrotic diseases. Additionally, CUR regulates angiogenesis (Macías-Pérez et al., 2019) and exerts protective effects on the lungs (Zhang et al., 2011), heart, liver, kidneys, and other organs by modulating various transcription factors, inflammatory mediators, protein kinases, growth factors, and cytokines (Figure 1).

## 2 Antifibrotic effects of curcumin

Before examining CUR’s effects across individual organ systems, several overarching limitations should be acknowledged. The field is characterized by a predominance of positive findings, with negative or null results rarely published—a pattern suggestive of considerable publication bias (Bhandary, 2024; Huang et al., 2024; Miao et al., 2021). Experimental designs vary substantially across studies, including differences in animal species and strains, fibrosis induction methods, CUR dosage and formulation, route of administration, treatment duration, and outcome measures. This heterogeneity precludes direct cross-study comparisons and meta-analyses (TV et al., 2022). Furthermore, most studies lack rigorous methodological safeguards, such as sample size calculations, randomization, blinding of investigators, or adherence to standardized reporting guidelines (Hajimirzaei et al., 2024), potentially introducing bias and limiting reproducibility. Therefore, the mechanistic insights derived from these studies should be interpreted with caution.

### 2.1 Respiratory system

#### 2.1.1 Pulmonary fibrosis

Pulmonary fibrosis (PF) is a chronic, progressive disease characterized by irreversible damage to lung structures. Its pathological features include inflammatory cell infiltration, excessive proliferation of fibroblasts, accumulation of fibrotic tissues (Chang et al., 2020; Macías-Pérez et al., 2019; Tyagi et al., 2016), abnormally increased collagen content, and remodeling of the extracellular matrix deposition (ECM)(Chang et al., 2020; Fernandez et al., 2012). Clinically, PF is highlighted by chronic dry cough and dyspnea and can progress to respiratory failure and death due to worsening respiratory function. The pathogenesis involves multiple signaling pathways, among which TGF-β/Smad acts as the central fibrotic driver and NRF2, NF-κB, and AMPK play key modulatory roles. CUR exerts antifibrotic effects in PF by targeting these pathways. The following subsections detail each pathway and the evidence supporting curcumin’s therapeutic potential.

##### 2.1.1.1 AKT/NRF2/HO-1 pathway

**FIGURE 2:**
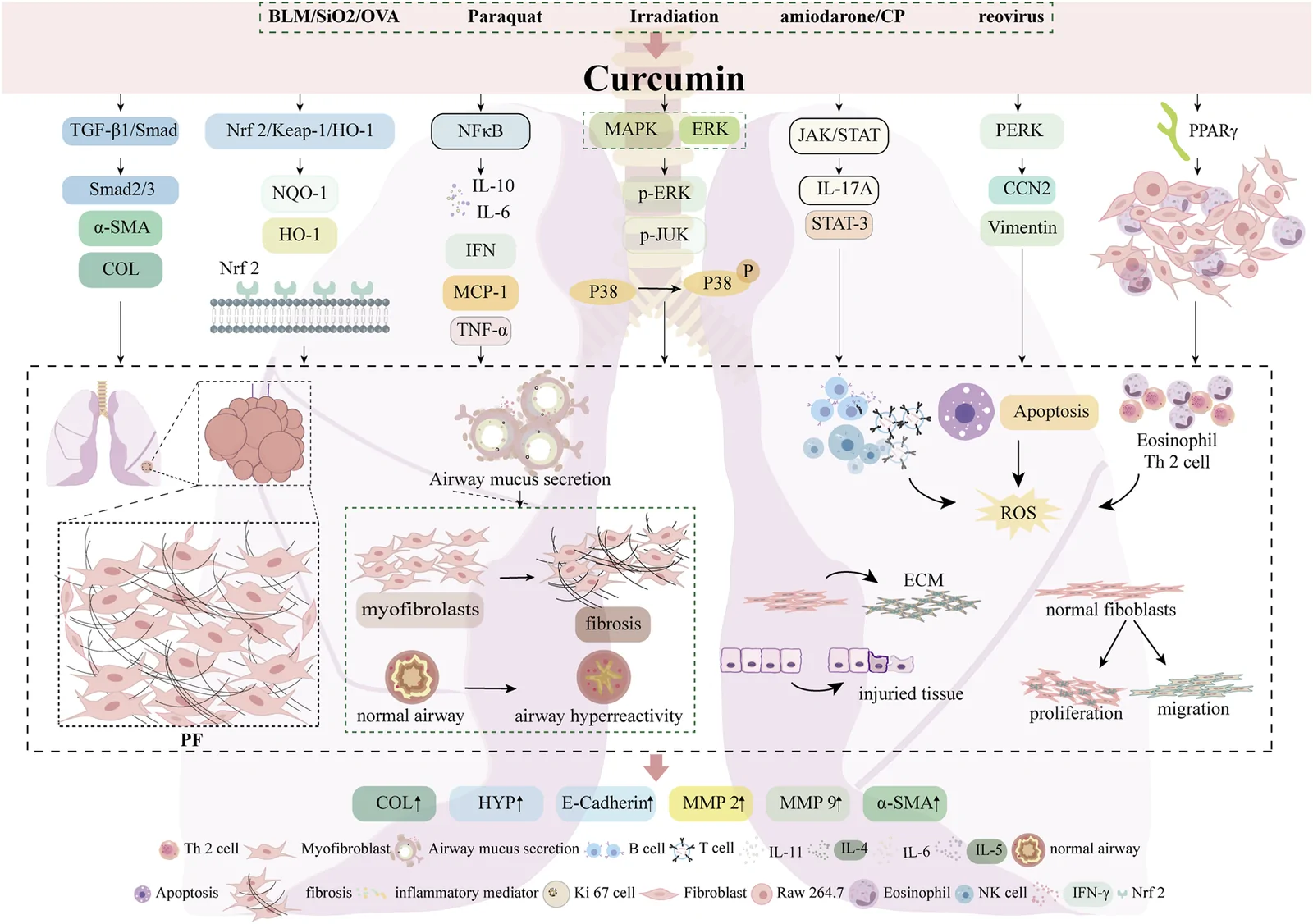
Multi-pathway regulatory network of curcumin against respiratory fibrosis. Curcumin exerts antifibrotic effects through coordinated modulation of multiple signaling pathways in pulmonary fibrosis, acute lung injury, and asthma models. The figure is organized in four layers: (1) inducers/insults [bleomycin (BLM), silica (SiO_2_), paraquat (PQ), irradiation, amiodarone, ovalbumin, and viral infection]; (2) core signaling pathways [transforming growth factor β (TGF-β)/Smad, NRF2/heme oxygenase 1 (HO-1), NF-κB, JAK/STAT, MAPK/extracellular-regulated protein kinase (ERK), p-ERK, and peroxisome proliferator-activated receptor-γ (PPARγ)]; (3) cellular events [fibroblast proliferation/migration/differentiation and alveolar epithelial cell apoptosis/epithelial–mesenchymal transition (EMT)/autophagy]; and (4) antifibrotic outcomes (reduced collagen deposition, hydroxyproline, matrix metalloproteinase (MMP)-2/9, inflammatory infiltration, and fibrotic area). The black solid arrows (→) indicate the flow of signaling and pathological events, the symbol (⊣) denotes inhibition or blockade, and the red upward arrows (↑) and green downward arrows (↓) exclusively indicate the upregulation and downregulation of molecular expression, respectively. The dashed lines indicate pathways with cell type-specific or context-dependent effects. For a comprehensive list of regulated molecules and corresponding references, please refer to Table 1.

CUR inhibits the expression of apoptosis-related factors, including reactive oxygen species (ROS) and cleaved caspase-3; promotes matrix metalloproteinase (MMP) secretion and AKT phosphorylation; and upregulates the expression of Bcl-2/Bax, NRF2, and HO-1 to protect against H_2_O_2_-mediated mouse lung mesenchymal stem cell (LMSC) injury in mice (Ke et al., 2020). Moreover, *in vitro* experiments using fibroblasts and alveolar epithelial cells derived from patients with advanced IPF confirmed that CUR upregulated p21 and p53 expression and promoted ROS release while inhibiting the expression of ACTA2, PCNA, collagen type I α1, CCND1, hypoxia-inducible factor 1α (HIF-1α), superoxide dismutase (SOD)2, and catalase (CAT) (Rodriguez et al., 2019). These inhibitory effects were observed only in normal lung fibroblasts. In contrast, the opposite result was found in A549 alveolar epithelial cells, indicating cell type-specific effects (Ke et al., 2020) (Figure 2; Table 1).

**TABLE 1:** Antifibrotic role of curcumin in respiratory fibrosis.

Disease	Animal/cell model	Inducer	Dosage and duration	Described effects	Reference
IPF	LMSCs	H_2_O_2_, 600 μM	Curcumin (2.5, 5, and 10 µM)	↑: MMP, Bcl-2/Bax, NRF2, HO-1, and p-AKT/AKT ↓: Apoptosis, ROS, and cleaved caspase-3	Ke et al. (2020)
PF/ALI	C57BL/6 male mice	BLM, 3 units/kg	Curcumin (75 mg/kg)	↑: MCH, platelet counts, uPA, and uPAR ↓: MCV and HGB counts, P-p53, p53, PAI-1, α-SMA, cleaved caspase-3, p-ERK, Ki-67-positive cell, IL-17A, PAI-1, and STAT3	Gouda et al. (2024)
ALI	Park’s strain mice	Paraquat, 50 mg/kg	Curcumin (5 mg/kg, i.n.)	↑: uPA and uPAR ↓: MMP-2, MMP-9, PAI-1, IL-17A, TNF-α, and IL-6	Muneesa et al. (2022)
ALI/LF	*In vitro*: A549	BLM (40 μg/mL)	Curcumin (20 µM)	↓: Ki 67, p-EGFR, and EGFR	Shaikh et al. (2020)
	*In vivo*: C57BL/6 mice		Curcumin (75 mg/kg)		
PF	*In vitro*: human embryonic WI-38 lung fibroblast cell	ET-1 and thrombin induce	Curcumin (5 µM)	↓: ET-1, α-SMA, CCN 2, vimentin protein, p-MAPK (ERK, JNK, and p38), p-ERK, and collagen IV	Chen et al. (2019a)
	*In vivo*: BALB/c mice	BLM, 0.05 U/50 µL	Curcumin (30 mg/kg)	↓: ET-1, α-SMA, CCN 2, vimentin protein, p-MAPK (ERK, JNK, and p38), p-ERK, and collagen IV	Chen et al. (2019a)
Lung fibrosis	*In vivo*: 57BL/6 mice	BLM, 0.1 mU/mL	Curcumin (200 mg/kg)	↑: Cathepsins K and L in the lungs and apoptotic cells ↓: Collagen deposition, TGF-β1, and cell proliferation and migration	Zhang et al. (2011)
	*In vitro*: HFL-1		Curcumin (3, 10, 20, and 30 µM)	↑: Cathepsins K and L, caspase-3, and Bax/Bcl-2 ↓: TGF-β1	Zhang et al. (2011)
IPF	*In vitro*: normal human fetal lung fibroblasts	TGF-β, 6 ng/mL	Curcumin (1, 5, 10, and 20 µM)	↓: Proliferation of lung fibroblasts, inflammation, collagen deposition, α-SMA, TGF-β1, p-Smad2, p-Smad3, and p-ERK/ERK	Smith et al. (2010)
	*In vivo*: C57BL/6 mice	BLM	Curcumin (300 mg/kg)	↓: HYP	Smith et al. (2010)
ALI/ARDS	Female CBA/J mice	Reovirus 1/L (10^7^ PFU) 15 mL in each nostril	Curcumin (50 mg/kg)	↑: p-p38 ↓: Procollagen I mRNA, IL-6, IL-10, IFNγ, MCP-1, α-SMA, E-cadherin, Tenascin-C, TGF-β RII, and P-p 65 NF-κB	​
PF	C57BL/6	BLM, 2 mg/kg	Curcumin (75 mg/kg), i.p.	↑:↑: uPA and uPAR ↓: MMP-2, MMP-9, and PAI-1	Muneesa et al. (2022)
PF	*In vivo*: ICR mice	BLM, 5 mg/kg or SiO_2_, 100 mg/kg	Curcumin (75 and 150 mg/kg)	↑: HGF (lungs and large intestine) and Klotho	Miao et al. (2021)
	*In vitro*: CCD-18Co cells, RAW264.7, and L929 cells		-	↑: PPARγ, CREB, HGF, PGD 2, and 15 d-PGJ 2	Miao et al. (2021)
IPF	*In vivo*: male SD rats	BLM	Curcumin LPMPs (5 mg)	↓: lung injuries, infiltration of pulmonary mesenchyme and the variation of alveoli, high lung deposition, macrophage uptake, HYP, collagen I, TNF-α, TGF-β1, NF-κB p65, and MMP-9	Hu et al. (2018)
PF	BALB/c mice	OVA, 50 μg	Curcumin (5 mg/kg)	↓: HYP, MMP-9, eotaxin, and α-SMA	Chauhan et al. (2016)
IPF	Primary human lung fibroblasts from a patient with IPF	​	Curcumin (10, 15, and 20 µM)	↑: KLF 10 and cell cycle arrest ↓: proliferation/viability of IPF fibroblasts and hsa-miR-6724-5 p	Chang et al. (2020)
ALI	Mice (Park’s strain)	PQ, 50 mg/kg, i.p., single dose	Curcumin (5 mg/kg)	↑: FITC B220 and PE CD4+ ↓: inflammatory cell count, reactive oxygen species (ROS), HYP, α-SMA, MMP-9, TIMP-1, FITC Gr1, and PE CD11	Tyagi et al. (2016)
PF	Female C57BL/6 mice	Irradiation, 13.5 Gy (8-min irradiation session)	1% or 5% (weight/weight, w/w) dietary curcumin	↑: HO-1 ↓: ROS and TNF-α	Lee et al. (2010)
	LLC cells or PMVEC	1.7 Gy/min	Curcumin (10 μM, 4 h)	↑: HO-1 ↓: ROS and TNF-α	Lee et al. (2010)
PF	​	​	​	↓: fibrosis, inflammation, oxidative, HYP, MPO, and MDA	Hanyu et al. (2023)
PF	*In vivo*: Wistar rats	BLM (0.75 U/100 g, 3, 5, 7, 14, and 28 days post-BLM)	Curcumin (300 mg/kg; 28 days)	↓: Total cell counts in BALF, ACE, AKP, superoxide anion, and nitric oxide	Durairaj Punithavathi (2000)
	*In vitro*: AM	BLM (3, 5, 7, 14, and 28 days)	-	↓: Total cell counts in BALF, ACE, AKP, superoxide anion, and nitric oxide	Durairaj Punithavathi (2000)
PF	Male Wistar rats	BLM (0.75 U/100 g body)	Curcumin (300 mg/kg)	↓: fibronectin level, total hexose, sialic acid, fucose, hexosamine, N-acetyl-β-d-glucosaminidase, and β-glucosidase	Durairaj et al. (2020)
PF	Fischer 344 rats	Amiodarone, 6.25 mg/kg	Curcumin (50, 100, and 200 mg/kg)	↓: MPO, TGF-β1, HYP, collagen I, c-Jun, TNF-α, and PMA	Punithavathi et al. (2009)
PF	*In vivo*: SD rats	BLM, 1.5 mg/kg	Curcumin (250 and 500 mg/kg; 28 days)	↓: collagen I, iNOS, and TGF-β1	Xu et al. (2007)
IPF	*In vitro*: pulmonary fibroblasts	Four patients with advanced IPF (IPF-F) and four normal lungs (NHLF)	Curcumin (20 µM)	↑: P21, P53, and ROS ↓: ACTA2, PCNA, collagen type I α1, CCND1, hypoxia-inducible factor 1α (HIF-1α), superoxide dismutase 2 (SOD2), and catalase (CAT), nuclear factor-like 2 (NRF2)	Rodriguez et al. (2019)
	*In vitro*: normal fibroblasts		Curcumin (20 µM)	↑: P21, P53, and ROS ↓: HIF-1 and SOD 2 -: CAT and NRF2	Rodriguez et al. (2019)
	*In vitro*: pulmonary epithelial		Curcumin (20 µM)	↑: P21, P53, and ROS ↓: HIF-1α, superoxide dismutase 2 (SOD2), CAT, and NRF2	Rodriguez et al. (2019)
IPF	*In vitro*: A549		Curcumin (20 µM)	↑: P21, P53, HIF-1, NRF2, CAT -: ROS	Rodriguez et al. (2019)
PF	Male albino Wistar rats	Cyclophosphamide (150 mg/kg b.w., i.p.)	Curcumin (200 mg/kg)	↑: NO and GSH ↓: HYP, elastin, MPO, MDA, TGF-β1, IL-1β, histamine, LT-C4, total protein, and N-acetyl-β-D-glucosaminidase, fibroblastic cells proliferation, and collagen	Hamdy et al. (2012)
IPF	*In vitro*: macrophages	2.5 U/kg BLM; immune inhibitor BLZ-945, M1: 100 ng/mL of LPS; and M2: 10 ng/mL of recombinant mouse IL-4	Mn-curcumin metal– organic frameworks	↓: M1, M2, pCSF-1R, HYP, TGF-β1, IL-11, IL-6, TNF-α, and ROS	Hou et al. (2024)
Cystic fibrosis	*In vitro*: HFF and mouse macrophages (J774A.1) cell lines	*P.* *aeruginosa* 10^6^ PFU reovirus 1/L	Curcumin-loaded lipid-polymer hybrid nanoparticles (termed CG-HNPs)	↑: Solubility and bioavailability and CTCF against *P. aeruginosa* biofilm and against intracellular bacteria	Sadeghi Mohammadi et al. (2021)
Inflammation and fibrosis	Male SD rats	A single 18-Gy dose of thoracic irradiation	Curcumin (200 mg/kg)	↓: TGF-β1, CTGF, TNF-α, collagen, TNFR 1, COX-2, and NF-κB p65	Cho et al. (2013)
Cystic fibrosis	*In vivo*: bronchial epithelial cell, VA10	-	Curcumin (1 and 10 mg/mL)	↑: Transepithelial electrical resistance ↓: F-actin localization, E-cadherin, and claudin-1 -: Occludin	Benediktsdottir et al. (2015)
PF	*In vivo*: Wistar rats	Paraquat, 20 mg/kg	Curcumin (200 mg/kg)	↓: Smad4, Smurf 2, IL-4, and IFN-γ	Chen et al. (2019b)
PF	*In vitro*: mouse lung fibroblast cell	TGF-β2, 10 ng/mL	Curcumin (5, 25, and 50 µM)	↑: PPARγ ↓: PDGFR-β, α-SMA, and proliferation -: FGFR 1	Liu D. et al. (2016)
Lung pneumonitis and fibrosis	Male Wistar rats	15 Gy using a cobalt-60 gamma rays source	Curcumin (150 mg/kg)	↓: IL-4, IL-13, IL4, Ra1, DUOX1, and DUOX2	Amini et al. (2018)
Asthma	Mice	OVA + DBP (10 mg/kg)	Curcumin (5 mg/kg)	↑: NQO-1, HO-1, and CAT ↓: CD 11b+, vimentin, E-cadherin, α-SMA, NRF2, and MMP-9/TIMP-1	Singh et al. (2025)
IPF	*In vitro*: A549	BLM (3.5 μM)	Curcumin analog EF24	↑: ROS and LC 3B ↓: Myofibroblast markers (vimentin, α-SMA, and snail), mtDNA, TOM 20, P62, and VADC 1	Zhang et al. (2024)
	*In vitro*: ATII cells	BLM (3.5 μM)	Curcumin analog EF24	↑: PTEN ↓: SA-β-Gal, collagen I, TGF-β, IL-6, PAI-1, P21, SASP, p-AKT`mTOR`NF-κB	Zhang et al. (2024)
	*In vivo*: C57BL/6 mice	BLM (3.5 mg/kg)	Curcumin analog EF24 (20 mg/kg)	↑: PTEN ↓: HYP, collagen I, SA-β-Gal, P21, and α-SMA	Zhang et al. (2024)
	*In vivo*:C57BL/6 mice	TBI (5 Gy)		↑: PTEN ↓: HYP, collagen I,, SA-β-Gal, P21, and α-SMA	Zhang et al. (2024)
IPF	*In vitro*: NIH 3 T3	TGF-β (5 ng/mL)	Curcumin (25 µM)	↓: Migration of fibroblasts, basement membrane collagen, fibronectin, MMP-2, MMP-9, inflammatory cells, disrupted alveolar structure, and thickened alveolar septa	Moideen et al. (2024)
	*In vivo*: C57BL/6 mice	BLM (2 mg/kg)	Curcumin (75 mg/kg)	↓: Collagen, fibronectin, MMP-2, and MMP-9	Moideen et al. (2024)
ALI	*In vivo*: C57/B6 male mice	IL-17A recombinant protein (1 µg)	Curcumin (75 mg/kg)	↑: uPA and uPAR ↓: IL-17A, JAK1, IL-27RA, HLA-DPA1, NFATC3, IL-2RB, GATA3, STAT3, p53, P-p53, PAI, and caspase-3	Gouda et al. (2024)
Asthma	BALB/c mice	OVA (50 µg) + Alum sensitization (i.p.) (days 0, 7, and 14); 1% OVA aerosol (days 19–22)	Curcumin (5 mg/kg)	↓: Inflammatory cell infiltration, ROS, NO levels, EPO, IgE level in serum, histamine level in BALF, MPO, HYP, and MMP-2 -: MMP-9, HDAC 1, H3acK9, and NF-kB p65	Chauhan et al. (2016)
Asthma	BALB/c mice	OVA (50 µg) + 0.2 mL saline containing 4 mg alum (i.p.) (days 0, 7, and 14); 1% OVA aerosol (days 19–22)	Curcumin (10 mg/kg)	↓: Oxidative stress (ROS and NO), MPO, EPO, IL-5, histamine, IgE, HYP, MMP-9, α-SMA, NF-κB p65, p-p38, p-JNK, and HDAC8 -: ALT, AST, creatinine, and pa-1	Islam et al. (2023)

##### 2.1.1.2 IL-17A/JAK/STAT pathway

Bleomycin (BLM) promotes fibrin deposition by increasing the expression of plasminogen activator inhibitor-1 (PAI-1) and decreasing the expression of urokinase plasminogen activator (uPA) and its receptor (uPAR). In BLM-induced PF in C57BL/6 male mice, CUR targeted the regulation of IL-17A-mediated inflammation (Gouda et al., 2019); played a key role in regulating p53-mediated fibrinolytic system damage and apoptosis of alveolar epithelial cells (AECs); increased MCH and platelet counts while decreasing the percentage of monocytes, lymphocytes, and granulocytes, as well as MCV and HGB levels; restored uPA and uPAR expression (Muneesa et al., 2022); inhibited p53 expression and its phosphorylation; and downregulated the expression of α-smooth muscle actin (α-SMA), cleaved caspase-3, Ki-67-positive cells, PAI-1, and STAT3 (Figure 2; Table 1).

##### 2.1.1.3 JNK signaling pathway

CUR not only targets and regulates the p53-mediated fibrinolytic system to restore normal expression of the fibrinolytically active components uPA, uPAR, and PAI-1 but also exerts anti-inflammatory and antifibrotic effects in models of acute lung injury (ALI) and PF induced by paraquat (PQ) and BLM by reducing the expression of the key tissue remodeling enzymes in PF, namely, matrix metalloproteinase-2 (MMP-2), and MMP-9 (Muneesa et al., 2022) (Figure 2; Table 1).

##### 2.1.1.4 p-ERK

Activation of p-ERK by ET-1 or thrombin induces lung fibroblast differentiation and accelerates PF development. CUR reduced the expression of α-SMA, CCN2, collagen IV, and vimentin proteins in lung tissues; decreased the levels of phosphorylated MAPK and p-ERK in WI-38 cells; and inhibited PF directly. Furthermore, CUR suppressed the ET-1 or thrombin-induced, concentration-dependent inhibition of CCN2, α-SMA, and vimentin proteins (Chen et al., 2019) (Figure 2; Table 1).

##### 2.1.1.5 TGF-β1

In models of IPF, including C57BL/6 mice, Sprague Dawley (SD) rats, and human fetal lung fibroblast 1 (HFL-1) cells, CUR induced the activation of lung tissue proteases K and L, reduced the proliferation and migration of lung fibroblasts, accelerated apoptosis, and reduced the production of hydroxyproline (HYP) and collagen, as well as inhibited the TGF-β1/Smad signaling pathway (Smith et al., 2010; Zhang et al., 2011; Liu D. et al., 2016). Additionally, CUR suppressed inducible nitric oxide synthase (iNOS) overexpression and reduced inflammatory damage and fibrosis (Xu et al., 2007) (Figure 2; Table 1). In another study, intranasal administration of 10^7^ PFU of reovirus induced ALI in female CBA/J mice; CUR treatment reduced the phosphorylation of NF-κB p65 and infiltration and expression of IL-6, IL-10, IFN-γ, and monocyte chemoattractant protein-1 (MCP-1) throughout the lung tissues and downregulated TGF-β1 receptor II, α-SMA, and tenascin-C expression through the inhibition of the TGF-β1 signaling pathway (Avasarala et al., 2015) (Figure 2; Table 1).

##### 2.1.1.6 Other pathways

CUR inhibited BLM- or silica (SiO_2_)-induced activation of PPARγ and CREB by 15 d-PGJ_2_ in a dose-dependent manner and increased hepatocyte growth factor (HGF) expression in primary mouse fibroblasts (Liu D. et al., 2016), macrophages, CCD-18Co fibroblast cells, and RAW264.7 monocyte–macrophage cells but not in primary colonic epithelial cells (Miao et al., 2021) (Figure 2; Table 1). In amiodarone- and cyclophosphamide (CP)-induced PF in Fischer 344 rats and male albino Wistar rats, CUR suppressed myeloperoxidase (MPO) activity and HYP levels in lung tissue (Hamdy et al., 2012); reduced TGF-β1 expression, collagen I, c-Jun, and elastin; and decreased lipopolysaccharide (LPS)-stimulated tumor necrosis factor alpha (TNF-α) release and phorbol myristate acetate (PMA)-stimulated superoxide production (Punithavathi et al., 2009). Treatment of BLM-stimulated lung macrophages with manganese–CUR metal–organic frameworks (MOFs) inhibited M1-to-M2 polarization and decreased IL-11, IL-6, and ROS (Hou et al., 2024). CUR-loaded large porous microparticles (CURLPMPs) attenuated BLM- and OVA-induced lung injury; reduced interstitial infiltration and alveolar degeneration; and downregulated eotaxin, α-SMA, collagen I, and MMP-9 (Chauhan et al., 2016). Both CUR nanomaterials and CURLPMPs inhibited the expressions of HYP, TGF-β1, and TNF-α, whereas CUR alone reduced NF-κB p65 expression (Hu et al., 2018) (Figure 5; Table 1). In female C57BL/6 mice exposed to 13.5 Gy irradiation for 8 min and in porcine proximal tubular epithelial (LLC) and pulmonary microvascular endothelial cells (PMVECs) treated with 1.7 Gy/min, CUR increased HO-1 levels in primary lung endothelial cells and fibroblasts, thereby blocking radiation-induced ROS and TNF-α (Lee et al., 2010) (Figure 2; Table 1). A meta-analysis encompassing 27 publications and 29 studies (396 animals) demonstrated that CUR exerts anti-inflammatory, antioxidant, and antifibrotic effects, reducing HYP, MPO, and malondialdehyde (MDA) levels (Hanyu et al., 2023). In male Wistar rats exposed to 15 Gy from a cobalt-60 gamma ray source, CUR downregulated IL-4, Ra1, DUOX1, and DUOX2 expression, attenuating irradiation-induced lung pneumonia and fibrosis (Amini et al., 2018). In SD rats with thoracic irradiation-induced PF at 18 Gy, CUR inhibited TGF-β1, connective tissue growth factor (CTGF), TNF-α, collagen deposition, and NF-κB p65 nuclear translocation and reduced the increase of TNFR 1 and cyclooxygenase-2 (COX-2) (Cho et al., 2013). CUR also suppressed total cell counts and the inflammatory response both *in vivo* and *in vitro*. In BLM-induced increase in bronchoalveolar lavage fluid (BALF), CUR decreased HYP, fibronectin, TNF-α, superoxide anion, and NO production by alveolar macrophages (Durairaj Punithavathi, 2000) and inhibited complex carbohydrates and glycosidases, such as total hexose, fucose, and N-acetyl-β-d-glucosaminidase in fibrotic lungs (Durairaj et al., 2020). Furthermore, in a study using primary human lung fibroblasts from an 83-year-old male patient with IPF, CUR treatment resulted in 13 downregulated and 57 upregulated microRNAs. Specifically, CUR reduced hsa-miR-6724-5p levels, leading to increased KLF10 expression, which induced cell cycle arrest and subsequently attenuated the proliferation and activity of IPF fibroblasts (Chang et al., 2020) (Figure 2; Table 1).

Accumulating evidence supports the antifibrotic activity of CUR in PF models. However, the majority of studies used acute BLM-induced injury rather than chronic, progressive disease paradigms and seldom assessed therapeutic efficacy in established fibrosis (Miao et al., 2021; Yu et al., 2025). Further limitations pertain to the considerable variability in administration routes, accompanied by a paucity of comparative bioavailability data (Jin et al., 2025), insufficiently powered validation of cell type-specific effects (Cheng et al., 2024), and substantial interspecies disparities that impede clinical translation (Yu et al., 2025).

#### 2.1.2 Acute and chronic airway inflammation: acute lung injury and asthma

ALI and asthma are distinct but overlapping pulmonary pathologies characterized by inflammation, oxidative stress, and progressive airway remodeling that can culminate in fibrosis. ALI is characterized by acute inflammation, alveolar epithelial dysfunction, and subsequent activation of fibroblasts into myofibroblasts that deposit excessive collagenous ECM, potentially progressing to PF (Gouda et al., 2019; Tyagi et al., 2016). Asthma is a chronic inflammatory airway disease characterized by Th2 cell-mediated eosinophilic infiltration, ROS production, and subepithelial fibrosis. Despite these differences in disease kinetics and etiology, CUR has been evaluated in both settings for its anti-inflammatory and antifibrotic potential (Islam et al., 2023) (Figure 2; Table 1).

In ALI models, CUR has been shown to mitigate lung injury and fibroplasia triggered by PQ intoxication, as evidenced by reduced α-SMA, matrix metalloproteinase 9 (MMP-9) and tissue inhibitor of metalloproteinases 1 (TIMP-1) expression and decreased inflammatory cell infiltration, collagen deposition, ROS and HYP levels, and secretion of IL-4 and IFN-γ (Chen Yang et al., 2019). In a murine model of virus-induced ALI/acute respiratory distress syndrome (ARDS), CUR treatment reduced cellular infiltration, cellular infiltration, focal lymphocyte accumulation, inhibited intra-alveolar and interstitial fibrosis, and downregulated collagen, α-SMA, E-cadherin, TGF-β1, and TGF-β receptor II (TGF-βRII) expression; decreased serum liver enzymes; and reduced the pulmonary infiltration of polymorphonuclear (PMN) (GSH reductase (GR) 1+), T, natural killer (NK), and B cells, potentially via the modulation of p38, phosphorylated IFNγ, and MCP-1 expression. Further *in vitro* A549 and *in vivo* evidence indicates that CUR directly attenuated PQ intoxication by decreasing α-SMA and MMP-9 and inhibiting altered TIMP-1 expression, with intranasal administration emerging as a promising therapeutic strategy (Shaikh et al., 2020; Tyagi et al., 2016) (Figure 2; Table 1).

In asthma models, CUR attenuated hallmark features, including serum IgE, histamine level in BAL, eosinophil peroxidase (EPO), and MPO, while reducing ROS and NO and inflammatory cell infiltration (Islam et al., 2022). Mechanistically, curcumin suppressed MAPK pathway activation; reduced oxidative stress; and attenuated epithelial migration, collagen deposition, α-SMA, extracellular-regulated protein kinase (ERK) downregulation, JNK phosphorylation expression, and p38 inhibition, slowing down subepithelial fibrosis, which was achieved through the MAPK pathway (Islam et al., 2023), and did not affect hepatic aminotransferases, creatinine, and NF-kB p65. In the OVA + DBP replicative asthma model, CUR inhibited NF-κB; activated the NRF-2/Keap-1/HO-1 signaling pathway; reduced CD 11b + cells; lowered the expression of waviness-associated proteins and α-SMA; and restored E-cadherin, the MMP-9–TIMP-1 ratio, and antioxidant enzyme levels (Singh et al., 2025) (Figure 2; Table 1). Despite consistent anti-inflammatory and antifibrotic activities in ALI and asthma models, the evidence base is critically constrained by model selection that poorly recapitulates human disease heterogeneity (Lelli et al., 2017), prophylactic dosing schedules that do not address therapeutic efficacy after disease onset (Huang et al., 2024), incomplete pharmacokinetic characterization, and insufficient mechanistic validation (Harshika et al., 2025). Furthermore, the absence of systematic investigations into cell type-specific effects, the lack of independent replication (Huang et al., 2024), publication bias against negative results, and considerable species differences collectively undermine translational confidence.

### 2.2 Digestive system

**FIGURE 3:**
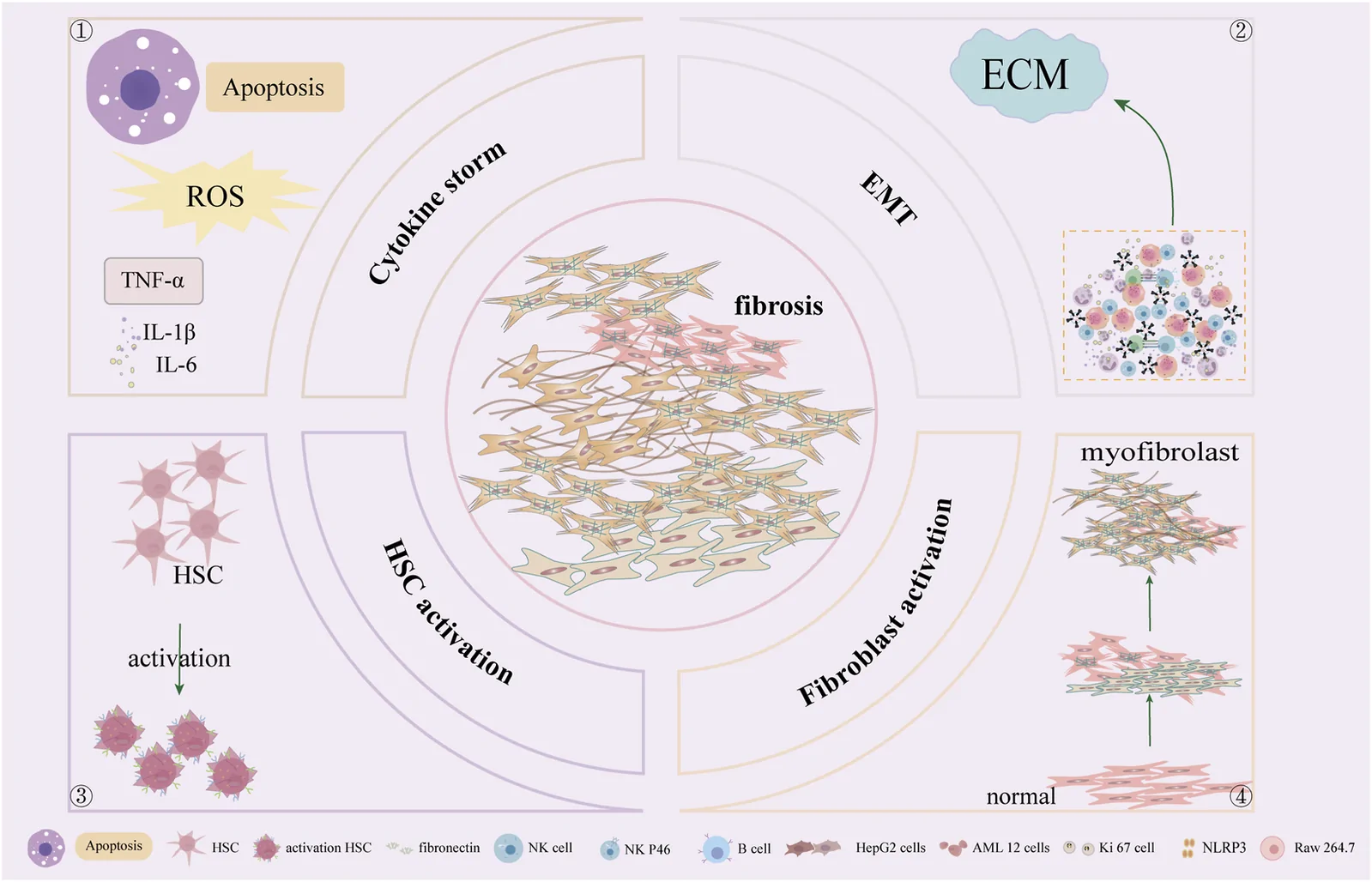
Central role of hepatic stellate cell (HSC) activation in liver fibrosis. Under normal conditions, HSCs remain quiescent. Upon liver injury or inflammatory stimulation, elevated levels of reactive oxygen species (ROS) and pro-inflammatory cytokines (e.g., TNF-α, IL-1β, and IL-6) induce HSC activation. Activated HSCs transdifferentiate into myofibroblasts, which overproduce the extracellular matrix (ECM), leading to fibrosis. Additionally, epithelial–mesenchymal transition (EMT) may further promote ECM deposition and fibrogenesis. In severe inflammation, a cytokine storm can exacerbate HSC activation and fibrosis progression. Arrows indicate regulatory or transformational relationships.

This section systematically elucidates the protective effects and multi-target molecular mechanisms of curcumin against fibrotic diseases in the digestive system, including the liver, oral cavity, and pancreas. Curcumin simultaneously modulates multiple pathological processes, including oxidative stress, inflammatory response, apoptosis, autophagy, and epithelial–mesenchymal transition (EMT). Curcumin inhibits liver fibrosis by suppressing the inflammatory storm, blocking hepatic stellate cell (HSC) activation, reducing myofibroblast conversion, and reversing EMT, acting synergistically at multiple stages of the fibrotic process (Figure 3).

#### 2.2.1 Oral submucous fibrosis

Oral submucous fibrosis (OSF) is a potentially malignant oral disease characterized by a burning sensation upon spicy food intake and whitening or stiffening of the oral mucosa, leading to restricted mouth opening. Treatment focuses on alleviating the limitation in mouth opening and the burning sensation (Rai et al., 2019; Zhang Tan et al., 2021; Mahato et al., 2019).

**FIGURE 4:**
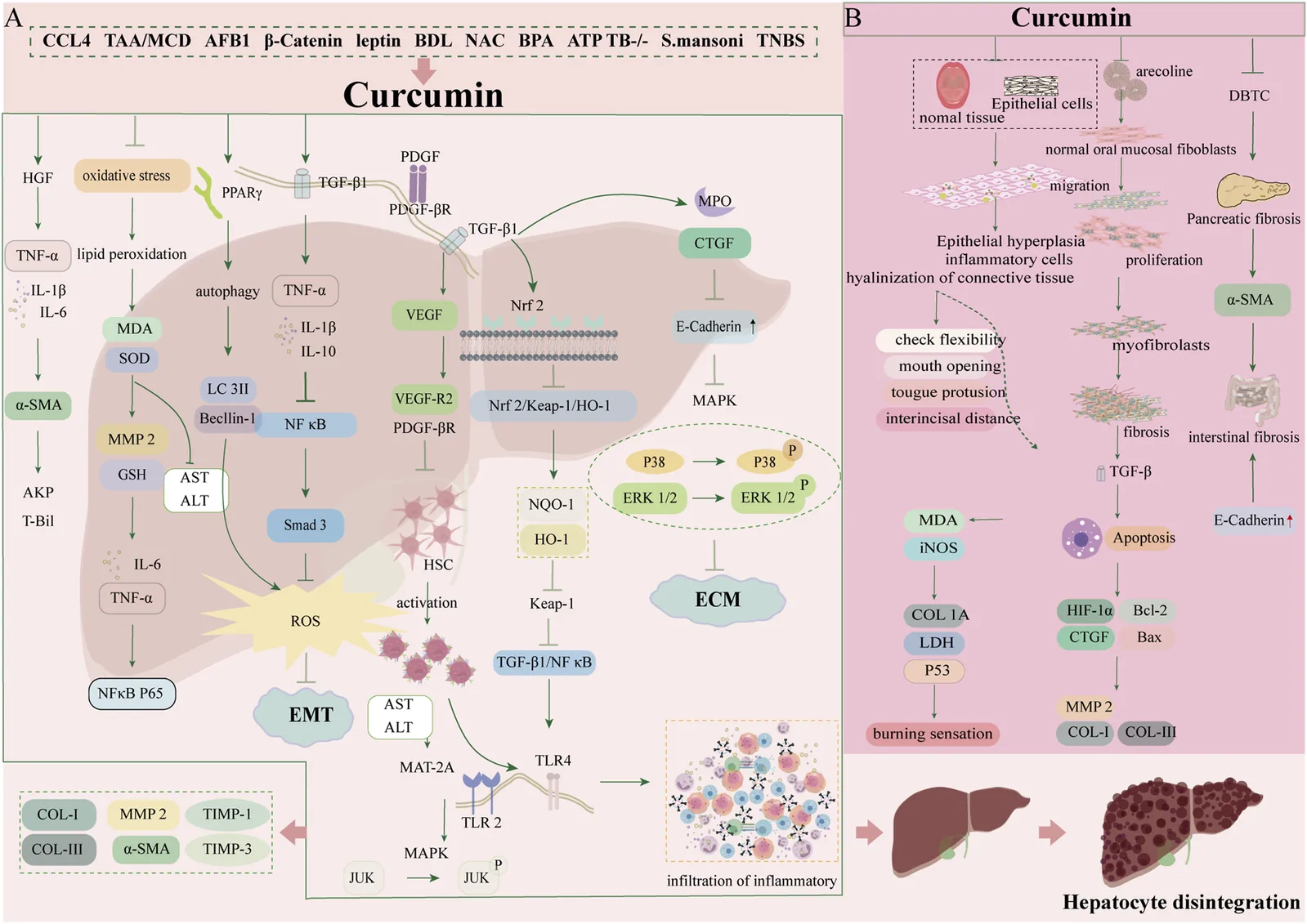
Antifibrotic mechanisms of curcumin in liver fibrosis, oral submucous fibrosis (OSF), and pancreatic fibrosis. **(A)** Curcumin protects against liver and pancreatic fibrosis via multiple signaling pathways. Curcumin activates NRF2/Keap-1/heme oxygenase 1 (HO-1) and peroxisome proliferator-activated receptor-γ (PPARγ); inhibits MAPK/NF-κB/transforming growth factor β1 (TGF-β1)/Smad3 axes; reduces oxidative stress [reactive oxygen species (ROS) and malondialdehyde (MDA)], inflammation (TNF-α and IL-6), and fibrosis markers (α-SMA and collagen IA); and modulates apoptosis (Bax/Bcl-2) and autophagy (Beclin-1). These effects suppress hepatic stellate cell (HSC) activation, epithelial–mesenchymal transition (EMT), extracellular matrix (ECM) deposition, and hepatocyte disintegration. These mechanisms have been validated in models including carbon tetrachloride (CCl_4_), thioacetamide (TAA), aflatoxin B1 (AFB1), bile duct-ligated (BDL), and others. Detailed molecular targets and clinical evidence are summarized in Supplementary Table S1. **(B)** Antifibrotic mechanisms of curcumin in OSF and pancreatic fibrosis.

In 40 patients with OSF treated with CUR gel or paste (Chandrashekar et al., 2021; Rai et al., 2019) and in 9 other studies involving 1,295 patients (sample sizes: 40, 30, 119, 60, 34, 30, 90, 685, 60, and 147) (Adhikari et al., 2022; Ahmad et al., 2021; Brignardello-Petersen, 2020; Deepak et al., 2021; Hazarey et al., 2015; Nerkar Rajbhoj et al., 2021; Piyush et al., 2018; Rai et al., 2019; Shao et al., 2024; Yadav et al., 2014), three month treatment with CUR tablets (300 mg once daily) improved mouth opening, tongue protraction, and buccal flexion and reduced burning sensation (Gupta et al., 2016). Another study on oral CUR reported reduced expression of p53, TGF-β, and iNOS expressions in 25%, 32.1%, and 32.1% of the samples, respectively. Multiple clinical trials have confirmed that CUR improves mouth opening, reduces burning sensation, decreases epithelial proliferation and inflammatory cell infiltration, and prevents connective tissue hyalinization (Al-Maweri, 2019; Alok et al., 2015; Rai et al., 2021) (Figure 4; Supplementary Table S1). In 11 further studies (428 patients), various CUR formulations improved tongue protrusion (Rai et al., 2021); increased mouth opening, elevated serum superoxide dismutase, iNOS, and vitamins C and E; decreased MDA and 8-hydroxydeoxyguanosine; reduced burning sensation; and caused no hepatorenal toxicity. Mechanistically, betulin directly damages the oral mucosa, and arecoline induces the proliferation and migration of normal oral mucosal fibroblasts. CUR promotes apoptosis, inhibits the HIF-1α/TGF-β/CTGF pathway; upregulates MMP-2 and Bax; and downregulates HIF-1α, TGF-β, CTGF, Bcl-2, and collagen type I/III α1, thereby attenuating OSF (Zhang Tan et al., 2021) (Figure 4; Supplementary Table S1). Clinical studies remain encouraging but are methodologically limited by small sample sizes, lack of blinding, heterogeneous outcome measures, short follow-up, and inconsistent diagnostic criteria with infrequent histopathological confirmation.

#### 2.2.2 Liver fibrosis

The liver is a central organ for energy metabolism, and liver fibrosis is closely associated with metabolic disorders (Wu Zhang et al., 2016). During liver fibrosis progression, PTEN methylation loss or gene deletion in liver tissues and activated HSCs in carbon tetrachloride (CCl_4_)-treated rats led to aberrant activation of the PI3K/AKT and ERK pathways, which inhibited HSC proliferation and induced HSC apoptosis (Geng et al., 2024; Zheng et al., 2014). HSC activation, a critical step in liver fibrosis, requires DNA methylation remodeling (Hu et al., 2020), and NRF2 has been identified as a potential target for antihepatic therapy (Lu et al., 2017). Cirrhosis results from uncontrolled fibrogenesis of hepatic sinusoids (Macías-Pérez et al., 2019; Zhang et al., 2014). The AMPK pathway exerts protective effects by promoting PGC-1α expression, enhancing PPARγ activity and SOD2 transcription, regulating oxidative stress and autophagy, activating PPARα to reduce ROS, and blocking mTOR-dependent signaling to increase autophagic flux (Kong et al., 2020; Wu Zhang et al., 2016; Zhai et al., 2015). These actions collectively reduce collagen fibrils, liver fibrosis, collagen deposition, HYP, serum glutamic oxaloacetic transaminase (AST), ALT, MDA, and lipid peroxidation (Liu et al., 2025). Additionally, excess hepatic iron exacerbates non-alcoholic fatty liver disease (NAFLD) by increasing hepatocyte expansion, inflammation, and fibrosis (Yu et al., 2024) (Figure 3B; Supplementary Table S1).

##### 2.2.2.1 AMPK pathway

CUR upregulated PPARγ, LXRα, and SOD2, thereby reducing HSC activation and suppressing type I collagen and PGC-1α expression to inhibit liver fibrosis (Zhai et al., 2015) (Figure 3; Supplementary Table S1). Subsequent experiments confirmed that CUR activates autophagy to inhibit the TGF-β/Smad pathway and upregulates LC3, Beclin-1, AMPK, and PPARγ while downregulating Smads, mTOR, and ROS. Consequently, CUR inhibited EMT and decreased liver enzymes, HYP, vimentin, fibronectin, hyaluronic acid (HA), PCIII, collagen IV, and α-SMA in CCl_4_-induced liver fibrosis rats and TGF-β1-stimulated BNL CL.2 cells (Kong et al., 2020; Qin et al., 2018) (Figure 4; Supplementary Table S1). In thioacetamide (TAA)-induced liver fibrosis mice, CUR similarly lowered serum ALT/AST levels, α-SMA, TIMP-1, and collagen I; activated AMPK; blocked mTOR signaling; elevated pro-apoptotic proteins and reduced TNF-α and Bcl-2 (Wu Zhang et al., 2016). These findings were validated in HSC-T6 cells, showing that CUR regulates HSC survival and activation to inhibit liver fibrosis (Figure 3; Supplementary Table S1).

##### 2.2.2.2 Autophagic and apoptotic pathways

CUR modulates autophagy and apoptosis to alleviate liver fibrosis. In TAA-induced liver fibrosis rat models and N-acetylcysteine-stimulated HepG2 cells, CUR increased survival; upregulated LC3-II and Bcl-2; downregulated SOD, MDA, and SQSTM1 via autophagy; and reduced fibrosis (Elmansi et al., 2017; Stefanska, 2012; Wang et al., 2012) (Figure 3; Supplementary Table S1). In TAA-replicated liver fibrosis mice and TGF-β1-stimulated AML12 cells, CUR reduced hepatocyte necrosis/apoptosis, collagen accumulation, and liver injury; downregulated TNF-α, collagen I-α1, and Bcl-2; upregulated p53, Bax, and PCNA; decreased ALT/AST/glucose; and inhibited oxidative stress, inflammation, and RAGE-mediated HSC activation (Figure 3; Supplementary Table S1). CUR concentration-dependently induced apoptosis in HSCs. *In vivo*, CUR protected against MCD diet-induced NAFLD and TAA-induced liver fibrosis by suppressing inflammatory and inducing apoptosis of damaged hepatocytes (Vizzutti et al., 2010; Wang et al., 2012) (Figure 4; Supplementary Table S1). CUR also reversed BPA-induced liver injury and fibrosis (Elswefy and Wahba, 2026), restored pro-oxidant/antioxidant balance, decreased IL-1β/IL-10, increased hepatocyte B cells, decreased hepatic caspase-3 and MMP-9, and upregulated metalloproteinase-2 tissue inhibitor (TIMP-2) to ameliorate EMT (Figure 3; Supplementary Table S1). In male ICR mice, CUR sensitized NK cells, promoted NK migration into the fibrotic liver, and cleared senescent HSCs. It upregulated high mobility group protein A1 and the DNA damage marker (γ-H2AX) and inhibited Bcl-2, Bax, and α-SMA, but did not affect cleaved caspase 3/8/9 (Jin et al., 2017; He et al., 2015) (Figure 3; Supplementary Table S1). In bile duct-ligated (BDL)-induced cholestatic liver fibrosis rats, CUR ameliorated COX-2, NF-κB, MDA, and HYP; increased glutathione (GSH) and Bcl-2; attenuated reduced liver enzymes and bilirubin; and reduced collagen fiber deposition. In ABEM-treated rats, CUR inhibited serum lipids, AST, and LDH; inhibited caspase-3, Bax, and TNF-α; and reduced liver fibrosis (Barta et al., 2015; El Swefy et al., 2015; Huyut et al., 2022). In summary, CUR exerts antifibrotic effects in the liver via coordinated autophagic and apoptotic pathways (Figure 3; Supplementary Table S1).

##### 2.2.2.3 CB/JNK/NF-κB pathway

CUR reduced collagen deposition, pro-inflammatory cytokine expression, and liver enzymes; decreased HYP and α-SMA; and increased CB2 expression (Huang et al., 2016) (Figure 3; Supplementary Table S1). CUR inhibited HSC activation and liver fibrosis by affecting leptin-induced methionine adenosyl transferase (MAT) 2A expression and downregulating p-JNK and α-SMA (Lu Zhao et al., 2021). In another study, CCl_4_-induced liver cirrhosis in male golden hamsters was reversed by CUR, which improved glucose and total protein levels, reduced liver enzymes, increased NRF2/NF-κB mRNA and protein expression in hepatic inflammatory cells, decreased the fibrotic area, and decreased type I and III collagen secretion (Macías-Pérez et al., 2019). CUR alleviated liver fibrosis by modulating the CB/JNK/NF-κ B pathway (Huang et al., 2016; Lu Zhao et al., 2021) (Figures 3, 4; Supplementary Table S1).

##### 2.2.2.4 DNA methylation

Wu Huang et al. (2016) and Zheng et al. (2014) found that CUR suppressed cell proliferation and apoptosis, decreased liver enzymes, reduced DNA methyltransferase 3b by upregulating DNA methylation, decreased PTEN, downregulated α-SMA and collagen, inhibited ECM deposition and DNA methylation, and blocked epigenetic activation of HSCs (Figure 3; Supplementary Table S1).

##### 2.2.2.5 EMT

**FIGURE 5:**
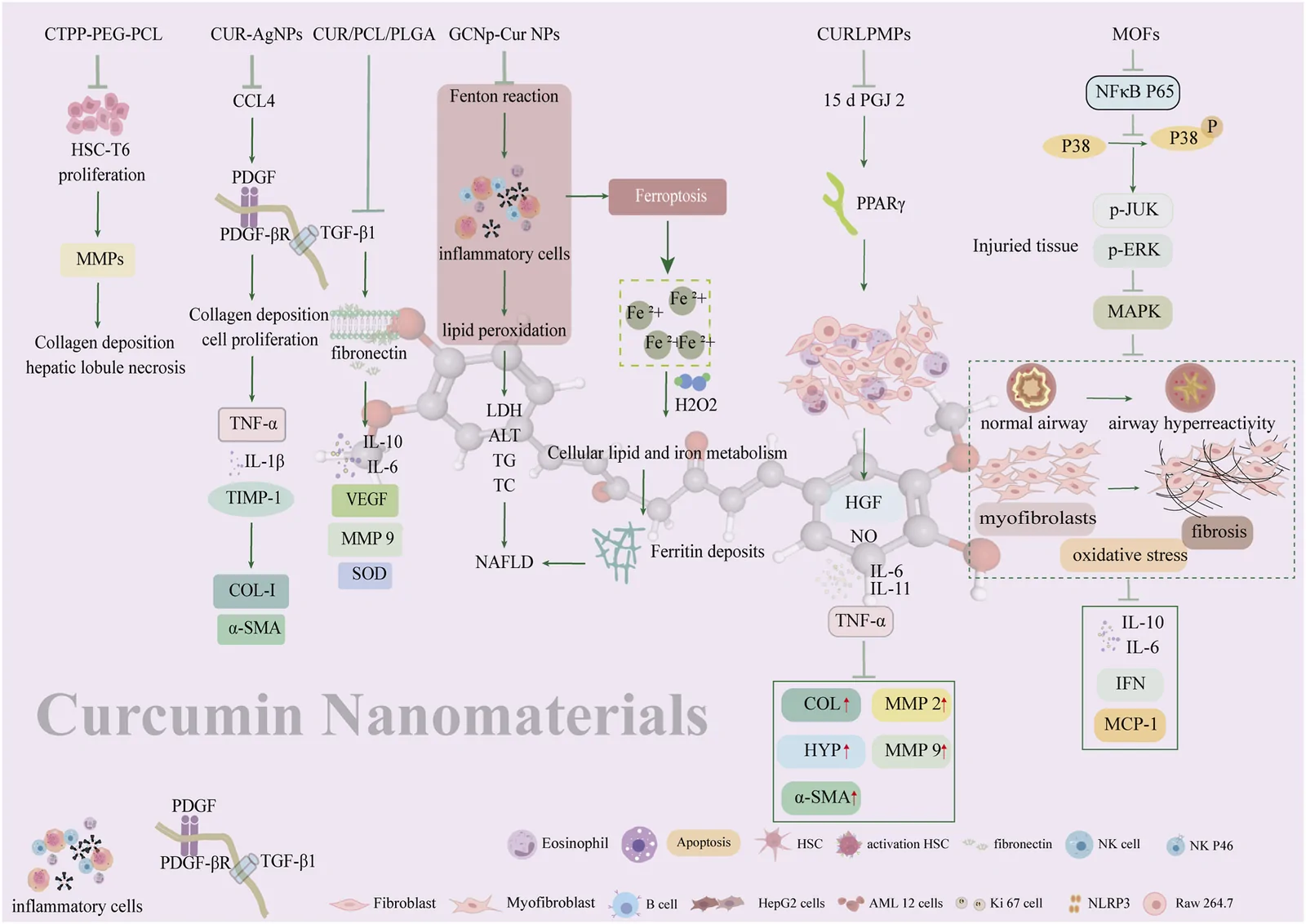
Curcumin (CUR) nanoparticles involved in the figures include 3-carboxypropyl-triphenylphosphonium bromide-poly(ethylene glycol)-poly(ε-caprolactone) (CTPP–PEG–PCL) micelles, CUR-silver nanoparticles (AgNPs), sophorolipid-coated CUR nanoparticles, GCNp-CUR NPs, CEHPNPs, CUR-loaded large porous microparticles (CURLPMPs), manganese–CUR metal–organic frameworks (MOFs), and liposome-encapsulated curcumin. CUR nanoparticles alleviate liver fibrosis by reducing oxidative stress [reactive oxygen species (ROS), H_2_O_2_, ferritin; activating superoxide dismutase (SOD)], suppressing inflammation (TNF-α, IL-1β, IL-6, and NF-κB P65; upregulating IL-10), inhibiting fibrosis [PDGF/transforming growth factor β1 (TGF-β1), collagen I, α-smooth muscle actin (α-SMA), and tissue inhibitor of metalloproteinase (TIMP)-1], modulating MAPK/p-extracellular-regulated protein kinase (ERK)/p-JAK pathways, and improving non-alcoholic fatty liver disease (NAFLD) markers (TG and TC), leading to reduced necrosis and myofibroblast activation.

CUR effectively inhibited CCl_4_-induced liver injury and liver fibrosis, decreased liver index, AST, alkaline phosphatase (ALP), HA, laminin, and type III procollagen levels; upregulated CBR2; downregulated CBR1, α-SMA, α1(I) procollagen, and fibronectin; and inhibited ECM deposition (Zhang et al., 2013; Zhang et al., 2012) (Figure 3; Supplementary Table S1). CUR reduced MMP-13 and collagen I collagen and inhibited mir-199/200 and their target genes (Hassan et al., 2012) (Figure 4; Supplementary Table S1). Algandaby Mm Fau–Al-Sawahli et al. (2016) prepared CUR-Zein nanospheres for enhanced hepatic targeting and antifibrotic activity, which decreased hepatic HYP, collagen I, TIMP-2 and TGF-β1; inhibited oxidative stress and MDA levels; increased serum albumin, SOD, CAT, GSH-Px, and MMP-2; and decreased liver enzymes, conferring hepatoprotection (Figure 4; Supplementary Table S1). In CCl_4_-induced adult SD rats, CUR promoted HGF, MMP-2, and pro-MMP-2 secretion; decreased liver function; inhibited TNF-α, IL-1b, and IL-6; and downregulated α-SMA (Wang et al., 2017) (Figure 3; Supplementary Table S1). Zhang Pan et al. (2021) constructed a CUR targeted micellar system 3-carboxypropyl-triphenylphosphonium bromide-poly(ethylene glycol)-poly(ε-caprolactone) (CTPP–PEG–PCL) complex] that enhanced cellular uptake and inhibited proliferation in HSC-T6 cells, exerted antifibrotic effects by reducing mitochondrial membrane potential and collagen deposition, and alleviated pseudolobular and hepatic lobular necrosis and cell proliferation (Figure 5; Supplementary Table S1).

##### 2.2.2.6 ERK

Eshaghian et al. (2018) and Zhao et al. (2014) modeled cholestatic fibrosis via BDL in rats. CUR improved liver histology; decreased liver enzymes and fibrosis-related factors; downregulated Rac1, Rac1-GTP, NOX1, SP1, leptin levels, SOCS3, STAT3, and insulin resistance; and increased IRS1 (Figure 4; Supplementary Table S1). Baghdasaryan et al., 2017 used Mdr2^−/−^ mice to model chronic cholangiopathy. Oral CUR reduced injury and cholestasis; upregulated PPARγ; downregulated Col1α2, α-SMA, p-ERK1/2, and VCAM-1; and reduced ALT, ALP, and HP but barely affected TGF-β-mediated NF-κB (p65) signaling (Figure 4; Supplementary Table S1). In HSCs and RAW264.7 cells stimulated with PDGF (platelet-derived growth factor), LPS (lipopolysaccharide), and IFN-γ (interferon-gamma), curcumin treatment significantly inhibited the expression of vascular endothelial growth factor (VEGF) and other pro-angiogenic signals, and reduced the phosphorylation of ERK1/2 and p38 MAPK, thereby attenuating the angiogenic properties of activated HSCs. Thus, CUR attenuates liver injury via ERK pathway modulation (Yao et al., 2026) (Figure 3; Supplementary Table S1).

##### 2.2.2.7 HIF-1α

In HFD-fed male ICR mice and dimethyl succinate-stimulated HSCs, CUR inhibited ROS production; prevented succinate- and cobalt chloride-induced HIF-1α upregulation; counteracted mitochondrial fatty acid oxidation (FAO); inhibited succinate dehydrogenase activity; reduced hepatic succinate accumulation; reduced NAFLD activity scores, fibrosis, collagen, and fibronectin gene expression; and blocked TGF-β1-mediated inflammation, thereby inhibiting HSC activation via the succinate/HIF-1α pathway (She et al., 2018) (Figure 4; Supplementary Table S1).

##### 2.2.2.8 Leptin signaling

Liver fibrosis and non-alcoholic steatohepatitis (NASH) are common in patients with obesity and diabetes with hyperglycemia, where hyperglycemia induces protein glycation to form advanced glycation end products (AGEs), a process accelerated by oxidative stress (Figure 3; Supplementary Table S1). Tang and Chen used *Lept* ^−/−^ and wild-type *Lept* ^+/+^ mice to isolate primary HSCs, which were stimulated with AGEs or leptin. CUR inhibited AGE gene expression and AGE-mediated HSC activation, decreased cellular oxidative stress, blocked leptin signaling, activated NRF2, and elevated GSH levels to counteract AGEs (Tang et al., 2014) (Figure 4; Supplementary Table S1).

##### 2.2.2.9 MAPK pathway

CUR decreased MAT 2B expression in HSCs, blocked p38 MAPK phosphorylation, reduced α-SMA and α1(1) collagen expression, and inhibited HSC activation in liver fibrosis (Hu et al., 2020) (Figure 3; Supplementary Table S1). In TAA-induced SD rats and TGF-β to stimulate HSC-LX 2 cells, dehydrozingerone (DHZ) promoted body weight gain, improved survival rate and total serum protein, decreased the liver–spleen index and liver enzymes, reduced oxidative stress and the mRNA/protein levels of inflammatory and fibrotic markers, upregulated E-cadherin, regulated the MAPK pathway, and reduced ECM deposition to prevent fibrosis (Sharma et al., 2022) (Figure 4; Supplementary Table S1).

##### 2.2.2.10 NRF2

CUR enhanced NRF2*,* NAD(P)H: quinone oxidoreductase 1 (NQO1), and HO-1 mRNA while reducing Keap-1 mRNA, exerting hepatoprotective effects (Kheiripour et al., 2021). It counteracts TGF-β1-induced PPARγ downregulation in HSCs and downregulates HA, laminin, PCIII, α-SMA, fibronectin, and α1(I) collagen. CUR also enhanced NRF2 expression, promoted its nuclear translocation and DNA binding, and induced the adipocyte-like transformation of HSCs through NRF2 activation (Zhang et al., 2012) (Figure 4; Supplementary Table S1). This transformation features increased lipid droplets and triglycerides; upregulation of PPARγ, NQO1, and HO-1; and reduced TNF-α and IL-6 secretion (Kheiripour et al., 2021). CUR further decreased liver enzymes, HYP, Keap-1 levels, and collagen deposition, thereby protecting liver tissue. Notably, NRF2 knockdown in human LX-2 cells abolished these protective effects (Lu et al., 2017) (Figure 4; Supplementary Table S1).

##### 2.2.2.11 PPARγ

In male C57BL/6 mice with CCl_4_-induced cirrhosis, CUR inhibited liver injury, pro-inflammatory cytokine production, and fibrosis; enhanced oxidized GSSG levels; upregulated PPARγ; suppressed pro-fibrotic factors; and induced HSC apoptosis via downregulation of IL-6, TNF-α, HYP, type I collagen α, fibronectin 1, and TGF-β (Bisht et al., 2011) (Figure 3; Supplementary Table S1). In HSCs, curcumin downregulated the Hedgehog (Hh) pathway components Patched and Smoothened, thereby blocking Gli1 nuclear translocation, DNA binding, and transcriptional activity. This led to the inhibition of DLK1, Shh, and PARP1 expression, downregulation of cyclins, and induction of mitochondrial apoptosis. Consequently, curcumin reduced HSC activation, suppressed liver fibrosis, and upregulated PPARγ, collectively alleviating liver fibrosis through Hh pathway inhibition (Lian et al., 2015) (Figure 3; Supplementary Table S1). PPARγ inhibits HSC-driven angiogenesis by downregulating PDGF-β receptor expression, suggesting that it is a molecular target to prevent vascular remodeling in liver fibrosis (Qiu et al., 2014; Liu D. et al., 2016).

##### 2.2.2.12 SHH pathway

In a mouse model of CCl_4_-induced liver fibrosis treated for 8 weeks, silver nanoparticles coated with a CUR-chitosan mixture and CUR-coated hyaluronic acid-polylactic acid nanoparticles (CEHPNPs) selectively inhibited liver fibrosis (Chen et al., 2016; Elzoheiry et al., 2022). These nanoparticles enhanced CUR membrane permeability and bioavailability in hepatocytes; reduced hepatic *COL1A1* and α-SMA expression; restored liver enzymes and histopathology; and decreased collagen deposition, cell proliferation, and the expression of TNF-α, IL-1β, IL-6, and TGF-β, as well as the positive rates of CD45, CD45^+^ leukocytes, F4/80^+^ macrophages, and CD11b^+^ monocytes (Zhao et al., 2018). CUR also decreased the fibrosis score, apoptosis index, and liver index; upregulated PPARγ; and downregulated collagen fibers and protein, HYP, α-SMA, HA, and laminin (Lian et al., 2015; Shu et al., 2009; Tu et al., 2012; Zhang et al., 2012; Zhang et al., 2014). Moreover, CUR attenuated inflammatory infiltration and inhibited TLR2, TLR4, and MCP-1 in the CCl_4_-induced cirrhotic rat model (Figure 5; Supplementary Table S1).

##### 2.2.2.13 TGF-β

Several studies confirm CUR’s hepatoprotective effects in liver fibrosis (Zhao et al., 2018). Lee et al. (2013) showed that dietary CUR inhibited DMN-induced liver fibrosis in rats by reducing oxidative stress and inflammation and improving liver function. CUR also inhibited HSC proliferation and induced apoptosis in ethanol-induced liver fibrosis (Chen et al., 2014; Lee et al., 2013; Zhao et al., 2012). Gowifel et al. (2020) reported that CUR attenuated fibrosis and cirrhosis via the NRF2/HO-1, NF-κB, and TGF-β/Smad3 pathways (Figure 4; Supplementary Table S1). Additionally, CUR upregulated Smad7, MMP-13, and MMP-2 (Hernández-Aquino et al., 2020; Ma et al., 2023; Morsy et al., 2011; Qun-yan Yao, 2012; Wu et al., 2010) but downregulated TGF-β1 and miR-122 in BDL-induced fibrosis (Gowifel et al., 2020; Nozari et al., 2020). Tawfeek et al. (2023) found that CUR nanofibers enhanced liver cell proliferation, differentiation, and migration while reducing inflammation and boosting antioxidants to alleviate CCl_4_-induced fibrosis (Abo-Zaid et al., 2020; Gowifel et al., 2020; Shu-Ju Wu, 2010) (Figure 3; Supplementary Table S1). CUR mitigates liver injury and fibrosis through various mechanisms.

##### 2.2.2.14 Wnt/β-catenin pathway

CUR inhibited β-catenin transactivation and DNA binding; decreased cytosolic β-catenin protein levels; and upregulated PPARγ, C/EBPα, RXRα, and RARβ, thereby affecting HSC activation and liver fibrosis (Zhang et al., 2016). In NASH, CUR blocked the leptin signaling pathway in HSCs; upregulated AMPK and PPARγ; downregulated leptin expression; inhibited oxidative stress and ECM deposition; decreased α-SMA, collagen deposition, and MMPs; and inhibited the apoptosis pathway (Cui et al., 2014; Tang, 2014) (Figures 3, 4; Supplementary Table S1).

##### 2.2.2.15 JAK2/NLRP3

In male Jinding ducks with aflatoxin B1-induced liver pyroptosis and fibrosis, inhibited growth, impaired liver structure and function, and activated the JAK2/NLRP3-mediated to induce pyroptosis and fibrosis (Cui et al., 2023). CUR promoted growth and restored body mass, reduced serum liver enzyme levels, decreased inflammatory cell infiltration and the expression of inflammatory cytokines and fibrosis-related proteins, ameliorated hepatocyte disruption, and inhibited the JAK2/NLRP3 signaling pathway (Figure 4; Supplementary Table S1).

##### 2.2.2.16 Other pathways

Wilson’s disease (WD) is an autosomal recessive copper metabolism disorder caused by ATP 7B gene mutations, characterized by hepatomegaly (Ho et al., 2022). Wai-In Ho et al. generated female *ATP7B* ^−/−^ mice as a WD model, CUR blocked high mobility group box 1 (HMGB1) translocation, reducing hepatic immune and macrophage infiltration, decreasing liver and spleen indices and liver enzymes, and attenuating p65 upregulation. Reduced HMGB1 translocation correlated with reduced splenic CD 11b^+^/CD 43^+^/Ly 6C^Hi^ monocyte and circulating pro-inflammatory cytokines, thus inhibiting hepatic inflammation and fibrosis (Figure 4; Supplementary Table S1). Yu et al. (2024) explored a CUR-loaded nanodelivery system-modulated GPX 4/GSH, which inhibited lipid peroxidation and inflammatory infiltration, restored redox balance, reduced free Fe^2+^, attenuated iron deposition and ferroptosis, induced lipid droplet lipoatrophy, decreased liver enzymes and lipids, improved lipid and iron metabolism, and inhibited ferritin deposition, delaying NAFLD (Figure 4; Supplementary Table S1). CUR also reduced collagen expression in NaOH-stimulated NIH/3T3 cells (Yu et al., 2019).

Despite extensive research, critical gaps remain in CUR’s role in liver fibrosis: lack of comparative studies across etiologies in a unified framework; short study durations precluding assessment of advanced cirrhosis and reversal; poorly characterized non-HSC contributions; unevaluated interference with physiological wound healing; and the absence of integrated analyses of signaling pathway interactions.

#### 2.2.3 Metabolic dysfunction-associated fatty liver disease

In two randomized controlled clinical trials (RCTs) involving 77 patients with NAFLD, CUR reduced energy intake, body weight, waist and hip circumference, BMI, liver fibrosis score, serum cholesterol, blood glucose, ghrelin, and physical activity (Saadati et al., 2019; Saeede Saadati, 2018). Another 12-week trial (250 mg/day of CUR) failed to significantly decrease fasting blood glucose, lipids, liver enzymes, and body weight or BMI but reduced waist circumference, blood pressure, steatosis, and fibrosis. In primary hepatocytes from 10 patients with Metabolic Dysfunction-Associated Steatotic Liver Disease, CUR-enriched foods promoted hepatic neo-lipogenesis, reduced inflammation and oxidative stress; decreased *Blautia producta*, 2-OG levels, body weight, fat mass, glucose, insulin, cholesterol, and triglycerides; and enhanced mitochondrial function (Safari et al., 2023; Sánchez-Tapia et al., 2024). Lukkunaprasit et al. (2023) analyzed 6 SRMAs and 16 RCTs and found that CUR decreased AST, ALT, hepatic steatosis, fasting glucose, BMI, and total cholesterol. In high-fat-diet-induced Wistar rats, seven month of CUR treatment yielded consistent results (Safari et al., 2023) (Figure 4; Supplementary Table S1).

#### 2.2.4 Periductal fibrosis

Charoensuk et al. (2016) induced acute and chronic periductal fibrosis (PDF) in hamsters by oral administration of 50 *Opisthorchis viverrini* metacercariae for 1 or 3 months. In the acute phase, CUR upregulated fibronectin and downregulated HYP, α-SMA, MMP-9, MMP-13, CTGF, and collagen types I and III. In the chronic phase, CUR upregulated MMP-13 and MMP-7 and downregulated HYP, α-SMA, ALT, MMP-9, MMP-13, and CTGF. Notably, in both phases, CUR had no effect on MMP-2 but consistently decreased HYP, α-SMA, ALT, MMP-9, CTGF, fibronectin, collagen types I and III, TGF-β, TNF-α, TIMP-1, TIMP-2, TIMP-3, and SH2-containing protein tyrosine phosphatase-1 (SHP-1), while upregulating CD10. *In vitro*, CUR reduced α-SMA, fibronectin, CTGF, and vimentin expression in peribiliary myofibroblasts from infected animals (Charoensuk et al., 2016) (Figure 3; Supplementary Table S1).

#### 2.2.5 Hepatobiliary fibrosis

Pinlaor et al. (2010) orally infected aged male Syrian golden hamsters with 50 *O*. *viverrini* metacercariae and treated them with CUR (1% curcumin in corn oil, w/w). CUR upregulated MMP-13, MMP-7, and IL-1β; downregulated collagen types I and III, TIMP-1/2, α-SMA, TNF-α, and HYP; and improved bile duct pathology, with no remarkable effect on MMP-2 or MMP-9 (Figure 4; Supplementary Table S1).

#### 2.2.6 Intestinal fibrotic stricture

*In vivo*, SD rats received 5% TNBS to induce fibrosis, followed by CUR treatment. CUR upregulated E-cadherin and PPARγ; ameliorated fibrosis, inflammatory cell infiltration, and collagen deposition; and downregulated α-SMA, TGF-β, fibronectin, and CTGF. In IEC-6 cells, CUR upregulated E-cadherin and PPARγ, downregulated α-SMA, and inhibited the progression of p-Smad3 signaling (Xu et al., 2017). In Caco-2 cells, CUR at <20 μg/mL maintained higher than 80% viability without cytotoxicity. Collectively, CUR improves intestinal fibrosis by inhibiting EMT (Figure 4; Supplementary Table S1).

#### 2.2.7 Pancreatic fibrosis

Lu et al. (2023) induced pancreatic fibrosis using dibutyltin dichloride. After 2 or 4 weeks of CUR treatment, it was observed that it ameliorated interstitial fibrosis and downregulated α-SMA expression (Lu et al., 2023) (Figure 4; Supplementary Table S1). However, studies on pancreatic and intestinal fibrosis remain sparse, with limited mechanistic insight and few studies addressing dose–response data and long-term outcomes.

#### 2.2.8 Schistosomiasis

In *Schistosoma mansoni*-infected mice, intraperitoneal CUR reduced granulomas and fibrous areas, decreased inflammatory cells and OV-6 percentage, and produced new hepatocytes (Alsulami et al., 2020). El-Agamy et al. (2011) found that CUR inhibited and reversed *S. mansoni*-induced liver fibrosis in male Swiss albino mice, reducing liver enzymes, bilirubin, albumin, and HYP (Figure 4; Supplementary Table S1).

### 2.3 Circulatory system

#### 2.3.1 Cardiac fibrosis

**FIGURE 6:**
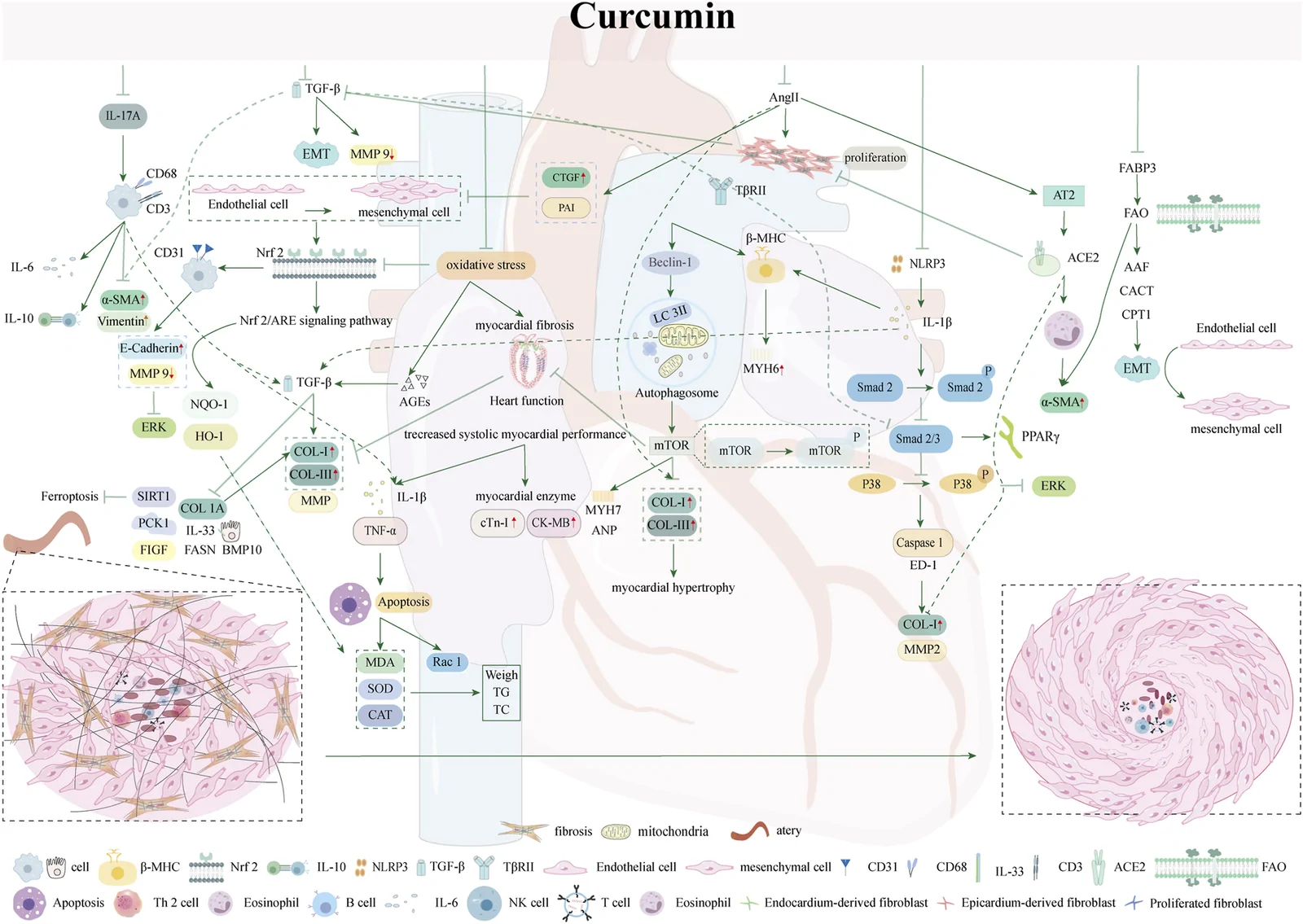
Antifibrotic role of curcumin (CUR) in circulatory system fibrosis. CUR exerts multifaceted antifibrotic effects in the cardiovascular system by modulating inflammation, oxidative stress, autophagy, and extracellular matrix remodeling. Upward arrows (↑) indicate upregulation or activation, whereas downward arrows (↓) indicate downregulation or inhibition. CUR suppresses pro-inflammatory cytokine production and reduces inflammatory cell infiltration. It inhibits the transforming growth factor β (TGF-β)/Smad signaling pathway, downregulates pro-fibrotic mediators, and attenuates myofibroblast differentiation and endothelial–mesenchymal transition (EndMT). Autophagy is modulated by the regulation of LC3-II, Beclin-1, and autophagosome formation, with context-dependent effects on cardiac fibroblasts and cardiomyocytes. Additionally, CUR activates the NRF2/heme oxygenase 1 (HO-1) antioxidant axis, reduces NADPH oxidase-mediated oxidative stress, and inhibits MAPK/extracellular-regulated protein kinase (ERK) phosphorylation. Collectively, these coordinated actions reduce collagen deposition, suppress fibrosis, and improve cardiac function.

Myocardial fibrosis is characterized by the excessive activation of cardiac fibroblasts (CFs) and collagen deposition, leading to interstitial remodeling. CFs drive the transition from cardiac compensation to decompensation and contribute significantly to heart failure mortality (Kanagala et al., 2019). Researchers have attempted to protect the myocardium by attenuating oxidative stress, inflammation, apoptosis, and fibrosis (Ji et al., 2016). CUR exerts antifibrotic and antiproliferative effects via the IL pathway, mTOR, PPARγ, and TGF-β1/Smad2/3 signaling pathways (Meng et al., 2014) (Figure 6; Table 2).

**TABLE 2:** Antifibrotic role of curcumin in circulatory system fibrosis.

Disease	Animal/Cell model	Inducer	Dosage and duration	Described effects	Pathway	Reference
DCM	Male Wistar rats	HFD + STZ (40 mg/kg)	Curcumin (100 and 200 mg/kg for 5 weeks)	↑: FS, EF, SOD, Bcl-2, f p-AKT, and p-GSK-3β ↓: Body weight, heart weight–body weight ratio, blood glucose, TG, TC, interventricular septal hypertrophy, IVSD, CK-MB, AST, LDH, IL-1β, MDA, TNF-α, glycogen lysis, cardiomyocyte hypertrophy, RAGE, gp91phox, p47phox, Rac1, NADP+/NADPH, Bax, and caspase-3	AKT/GSK-3β signaling pathway	Yu et al. (2012)
Cardiac fibrosis	Rats	ABE (26 mg/kg/day)	Curcumin (30 mg/kg for 4 weeks)	↓: Troponin I, total cholesterol, CK-MB, cardiac fibrosis, cardiac vessels, myofibril degeneration, karyolytic myocardiocytes, and fibrosis	CK-MB	Huyut et al. (2023)
Atrial fibrillation	Male SD rats	Ach (66 μg/mL)–CaCl_2_ (10 mg/mL) for 28 days	Curcumin (50 mg/mL for 24 days)	↓: AF duration, left atrial fibrosis, myocardial fibrosis, IL-17A, IL-1β, IL-6, TGF-β1, collagen type I α1, FASN, PCK1, BMP10, IL-33, and FIGF	IL-17	Yue et al. (2022)
MI	*In vivo*: Male C57BL/6 mice	Left anterior descending coronary artery ligation	Curcumin (50 and 100 mg/kg) for 4 weeks	↓: CD68+, CD3+, cardiac myofibroblast enrichment, collagen deposition, periostin, vimentin, and α-SMA	IL18–p-SMAD2/3 signaling pathway	Zhao et al. (2022)
	*In vitro*: Raw RAW264.7 cells	LPS 1 μg/mL	Curcumin (10 and 20 μM)	↑: IL-10 ↓: Proliferation, apoptotic, IL-6, IL-1β, TNF-α, and CD86+		
	*In vitro*: primary neonatal rat cardiac fibroblasts	TGF-β 10 ng/mL	Curcumin (20 μM)	↓: α-SMA, vimentin, periostin, and EDA–fibronectin, p-SMAD2/3, and IL-18		
Cardiac hypertrophy	Male SD rats	Isoprenaline (5 mg/kg/day)	Curcumin (200 mg/kg/day for 4 weeks)	↑: MYH6, β-MHC (MYH7B), and mTOR ↓: Cardiac hypertrophy, interstitial fibrosis, heart weight–body weight ratio, ANP, MYH7, collagen I, collagen III, LC3, Beclin-1, and p-mTOR -: BNP	mTOR/autophagy	Liu R. et al. (2018)
CF (chronic kidney disease)	*In vivo*: male SD rats	Remove the right kidney and select ligation of the posterior branch and the anterior segmental artery of the anterior branch	Curcumin (100 mg/kg for 5 weeks)	↓: Left kidney weight, proteinuria, high blood pressure, LV mass, interstitial fibrosis, cardiomyocyte hypertrophy, ED-1 (heart), NLRP3, caspase-1, IL-1β, TGF-β1, p-Smad2, collagen I, and β-MHC -: Glomerulosclerosis, urinary protein, blood urea nitrogen levels, ED-1 (kidney), and ASC	NLRP3	Bugyei-Twum et al. (2016)
DCM	*In vivo*: male SD rats	HFD + STZ (30 mg/kg)	Curcumin (20 mg/kg for 8 weeks)	↑: SOD, NRF2, NQO 1, and CAT ↓: MDA, TGF-β1, and collagen type I α2	NRF2/ARE pathway	Xiang et al. (2020)
CF	*In vitro*: HCFs	TGF-β1 10 ng/mL	Curcumin (20 µM)	↓: α-SMA, collagen I, collagen III, cyclin B, CDK1, p-Smad2/3, p38 MAPK, and p-ERK	p38 MAPK/ERK signaling pathway	Fang et al. (2018)
CF	*In vitro*: cardiac fibroblasts	TGF-β1 10 ng/mL	Curcumin (25 mM)	↓: Proliferation, migration, collagen, MMP-2, p-Smad2/3, p-AKT, AT1R, and p-ERK1/2	Smad/ERK	Chung (2014)
CF	*In vitro*: CFs from SD rats	TGF-β1 10 ng/mL	Curcumin (20 µM)	↓: α-SMA, collagen I, Smad2, p-Smad2, P38, and p-P38	Smad-2 and p38 MAPK signaling pathway	Liu L. et al. (2016)
CF	*In vitro*: cardiac ventricular fibroblasts	Ang II (10^−5^ mol/L) for 24 h	Curcumin (5, 10, and 20 µM)	↓: collagen I, collagen III, TGF-β1, MMP-9, and TIMP-1	TGF/MMP	Ma et al. (2016)
DCM	Male SD rats	HFD + STZ (35 mg/kg)	Curcumin (300 mg/kg for 16 weeks)	↑: Smad7 ↓: collagen I, collagen III, TGF-β1, TβR II, and p-Smad2/3	TGF/Smad/AMPK	Guo et al. (2017)
DCM	*In vitro*: HCFs	HG (30 mmol/L)	Curcumin (25 µM for 24 h)	↑: Smad7 ↓: collagen I, collagen III, TβR I, TβR II, and p-Smad2/3		
		TGF-β1 (5 ng/mL)	Curcumin (25 µM for 24 h)	↓: collagen I, collagen III, p-P38, AMPKα, and p-AMPKα -: Smad7		
CF	*In vitro*: HCFs	Ang II 0.1 μmol/L	Curcumin (5, 10, and 20 µM)	↑: PPARγ ↓: CTGF, PAI-1, collagen III, FN, TGF-β1, and p-Smad2/3	TGF-β1/Smad2/3	Meng et al. (2014)
CF	SHRs	-	Curcumin (100 mg/kg/day for 12 weeks)	↑: PPARγ ↓: SBP, blood Ang II concentration, heart weight, heart weight–body weight ratio, left ventricle weight–body weight ratio, CTGF, PAI-1, collagen III, and FN		
Myocardial fibrosis	Male SD rats	Ang II (500 ng/kg/min)	Curcumin (150 mg/kg/day for 4 weeks)	↑: AT 2, AT 2/AT 1, and ACE2 ↓: Arterial blood pressure, mean arterial pressure, AT 1, α-SMA, accumulated macrophages, proliferated myofibroblasts, TGF-β1, p-Smad2, p-Smad3, and collagen I	TGF-β1/Smads signaling pathway	Pang et al. (2015)
MI	*In vivo*: Male C57BL/6J wild-type mice	Permanent ligation of the left anterior descending coronary artery	Curcumin (100 mg/kg/day for 4 weeks)	↑: SIRT1 ↓: collagen I, collagen III, and TGF-β1	-	Ji et al. (2016)
	*In vitro*: HCFs	Ang II (1, 10, and 100 nM) for 48 h	Curcumin (5, 10, and 15 µM)	↑: SIRT1 ↓: collagen I, collagen III, TGF-β1, MMP-2, and MMP-9		
CF	*In vivo*: male SD rats	Isoproterenol hydrochloride (5.0 mg/kg/day)	Curcumin (150 and 300 mg/kg/day for 4 weeks)	↓: LVIDd, LVIDs, perivascular and cardiomyocyte fibrosis, fibroblast transformation, collagen I, collagen III, and α-SMA, -: EF	-	Ma et al. (2016)
Takayasu’s arteritis	TAK patients (n = 12) and healthy controls (n = 8)	​	Curcumin granules (60 g/day for 3 months)	↑: Fatty acid oxidation ↓: Inflammation cells infiltration, thickening, necrosis and elastic fiber breakage, FABP3, α-SMA, ECM, AAF, CPT1A, CACT, ATP production, and cell proliferation	-	Sifan Wu (2022)
Endothelial cell fibrosis	HUVECs	TGF-β1	Curcumin (5 and 10 μM)	↑: NRF-2, VE-cadherin, DDAH, and CD31 ↓: IL-1β, TNF-α, ADMA, vimentin, p-ERK1/2, EGFR, MMP-9, and α-SMA	EndMT and ERK1/2 pathway	Chen et al. (2020)

##### 2.3.1.1 AKT/GSK-3β signaling pathway

In a rat model of diabetic cardiomyopathy (DCM) induced by high-fat chow feeding combined with streptozotocin (STZ), CUR reduced systolic myocardial dysfunction, thereby alleviating myocardial dysfunction and fibrosis (MF), restoring abnormal metabolism and impaired myocardial enzymes, and attenuating oxidative stress (Figure 6; Table 2). CUR also reduced the inflammatory factors IL-1β and TNF-α and cardiomyocyte apoptosis; downregulated MDA, SOD, the NADP^+^–NADPH ratio, Rac1, and NADPH activity; attenuated dyslipidemia and AGES accumulation; decreased TG, TC, body weight gain, and the heart weight–body weight ratio, thereby delaying the progression of DCM (Yu et al., 2012) (Figure 6; Table 2). In another study, CUR activated the NRF2/ARE pathway, which alleviated oxidative stress by restoring MDA and SOD levels; protected myocardial morphology; increased NRF2, NQO1, and CAT expression; decreased TGF-β1, collagen deposition, troponin I, creatine kinase-MB, and total cholesterol; reduced cardiomyocyte lysis; and improved CF (Huyut et al., 2023; Xiang et al., 2020) (Figure 6; Table 2).

##### 2.3.1.2 IL pathway

In male SD rats, 28-day Ach–CaCl_2_ gavage induced atrial fibrillation (AF). CUR reduced AF duration and MF; decreased IL-17A, IL-1β, IL-6, and TGF-β1; and downregulated *COL1A1*, *FASN*, *PCK1*, *BMP10*, *IL33*, and *FIGF*, thereby treating AF via the IL-17 pathway (Yue et al., 2022). In male C57BL/6 mice, left anterior descending coronary artery ligation was used to model myocardial infarction (MI). CUR reduced CD68^+^/CD3^+^ positivity, fibroblast enrichment, and collagen deposition (Zhao et al., 2022). *In vitro*, CUR promoted IL-10 and downregulated pro-inflammatory factors in RAW264.7 and TGF-β-stimulated neonatal CFs, attenuating post-MI inflammation (Ji et al., 2016) (Figure 6; Table 2). Using the same MI model in male C57BL/6J mice (Yue et al., 2022), found that CUR reduced collagen I/III and TGF-β1; remarkably decreased collagen deposition; inhibited CF deposition proliferation, migration, and MMP expression; and restored SIRT1 in human CFs. Mechanistically, CUR downregulated the expression of waveform protein, α-SMA, and periosteal hyperplasia protein; achieved cardiomyocyte proliferation and apoptosis; and improved cardiac function, thereby inhibiting myocardial fibrosis by blocking macrophage–fibroblast crosstalk in the acute phase of MI and the IL18-P-SMAD2/3 signaling pathway in CF (Zhao et al., 2022) (Figure 6; Table 2).

##### 2.3.1.3 mTOR/autophagy

Cardiac hypertrophy (CH), characterized by cardiomyocyte growth and increased heart mass, is a major cause of heart failure. In an isoprenaline-induced CH model, CUR inhibited autophagy and activated mTOR, thereby alleviating CH and fibrosis. Mechanistically, CUR upregulated MYH6, β-MHC (MYH7B), and mTOR while decreasing the expression of ANP, MYH7, collagen I/III, LC3, Beclin-1, p-mTOR, and the heart weight–body weight ratio, with no effect on BNP (Liu R. et al., 2018) (Figure 6; Table 2).

##### 2.3.1.4 MAPK/Smad/ERK signaling pathway

CUR inhibited TGF-β1-mediated CF activity via multiple pathways: promoting G2/M-phase cell cycle arrest, downregulating cycle B and CDK1, and reducing CF differentiation and collagen deposition (Fang et al., 2018). CUR also modulated fibroblast proliferation and migration, reduced ECM deposition, and inhibited Smad/ERK and P38 MAPK signaling, thereby improving fibrosis (Cheng-Chih Chung, 2014; Liu D. et al., 2016) (Figure 6; Table 2). Studies in SD rats also found that CUR reduced the expression and phosphorylation of fibrotic CFs and fibrosis, suggesting that CUR attenuated AF-induced fibrosis by targeting CF activity and its signaling pathways. In a chronic kidney disease induced cardiac fibrosis model, Bugyei-Twum et al. (2016) found that CUR inhibited NLRP3 inflammasome activation; ameliorated cardiac fibrosis and diastolic dysfunction; reduced left kidney weight, left ventricular mass, interstitial fibrosis, and cardiomyocyte hypertrophy; decreased proteinuria; attenuated cardiac NLRP3 activation and IL-1β release; and downregulated multiple relevant genes all without affecting renal structure and function (Figure 6; Table 2).

##### 2.3.1.5 TGF

A series of *in vitro* and *in vivo* studies have shown that CUR inhibits angiotensin II-induced cardiac fibroblast proliferation, reduces collagen expression, downregulates multiple fibrosis-associated proteins, suppresses the TGF/Smad/AMPK signaling pathway, promotes collagen degradation, attenuates myocardial fibrosis by inhibiting fibrotic protein production and downregulating TGF-β1 and p-Smad2/3 while promoting PPARγ (Guo et al., 2017; Meng et al., 2014), and decreases myofibroblast numbers and fibroblast proliferation and differentiation (Guo et al., 2017; Meng et al., 2014; Pang et al., 2015). Overall, CUR exhibits promising antifibrotic effects across diverse experimental settings (Figure 6; Table 2). However, the following critical gaps remain: heavy reliance on pressure overload and ischemic models with limited exploration of diabetic, arrhythmic, or aging-related fibrosis; conflation of reparative versus reactive fibrosis; poorly resolved fibroblast heterogeneity and intercellular crosstalk; hindered translation due to inconsistent dosing, limited pharmacokinetics, and unaddressed clinical end points; and the absence of integrated analyses of inflammation–fibroblast–remodeling interplay.

#### 2.3.2 Vascular fibrosis

Takayasu’s arteritis (TAK) is a chronic progressive inflammatory disease that primarily affects large arteries and their branches and is histopathologically characterized by granulomatous vasculitis and fibrosis. Current treatment with glucocorticoid and immunosuppressive agents has failed to effectively block the progression of vascular fibrosis in TAK (Seyahi, 2018). Studies have shown that FABP3 expression is increased in the thickened outer membrane adventitia of affected vessels in TAK and positively correlates with serum levels of ECM components. Upregulation of FABP3 promotes the expression of FAO, a mitochondrial metabolic process that generates energy through the conversion of fatty acids into various metabolites. In a clinical observation study in 12 patients with TAK and 8 healthy controls, oral administration of CUR granules (60 g/day for 3 months) resulted in increased FAO levels; decreased the expression of α-SMA, ECM, AAF, CPT1A, and CACT in the blood; reduced ATP production; and diminished cell proliferation, thereby inhibiting vascular fibrosis (Sifan Wu, 2022) (Figure 6; Table 2).

#### 2.3.3 Endothelial cell fibrosis

Endothelial–mesenchymal transition (EndMT) is one of the mechanisms by which myofibroblasts are generated in fibrotic tissues or organs and is characterized by the loss of adhesion and polarity between endothelial cells, conversion to mesenchymal cells, acquisition of a mesenchymal phenotype, and an increase in cell migration and differentiation. In human umbilical vein endothelial cells (HUVECs) stimulated with TGF-β1, CUR treatment upregulated NRF2, VE-cadherin, DDAH, and CD31 while decreasing the phosphorylation of MMP-9 and ERK1/2, thereby attenuating endothelial cell fibrosis (Chen et al., 2020) (Figure 6; Table 2). Collectively, these findings indicate that CUR can intervene in TAK-related vascular fibrosis through multiple pathways, providing experimental and clinical evidence for its potential therapeutic application in TAK. However, existing studies on arterial and endothelial fibrosis, particularly in Takayasu’s arteritis, are limited to small patient cohorts and lack mechanistic validation in experimental models. The generalizability of these findings to other vasculopathies remains uncertain, and the long-term safety of curcumin in the context of vascular remodeling has yet to be established.

### 2.4 Urinary system

Renal fibrosis is characterized by disruption of TGF-β1-mediated EMT transformation and PI3K/AKT signaling pathways, as well as activation and proliferation of interstitial fibroblasts.

#### 2.4.1 APPL1/AKT signaling pathway

**FIGURE 7:**
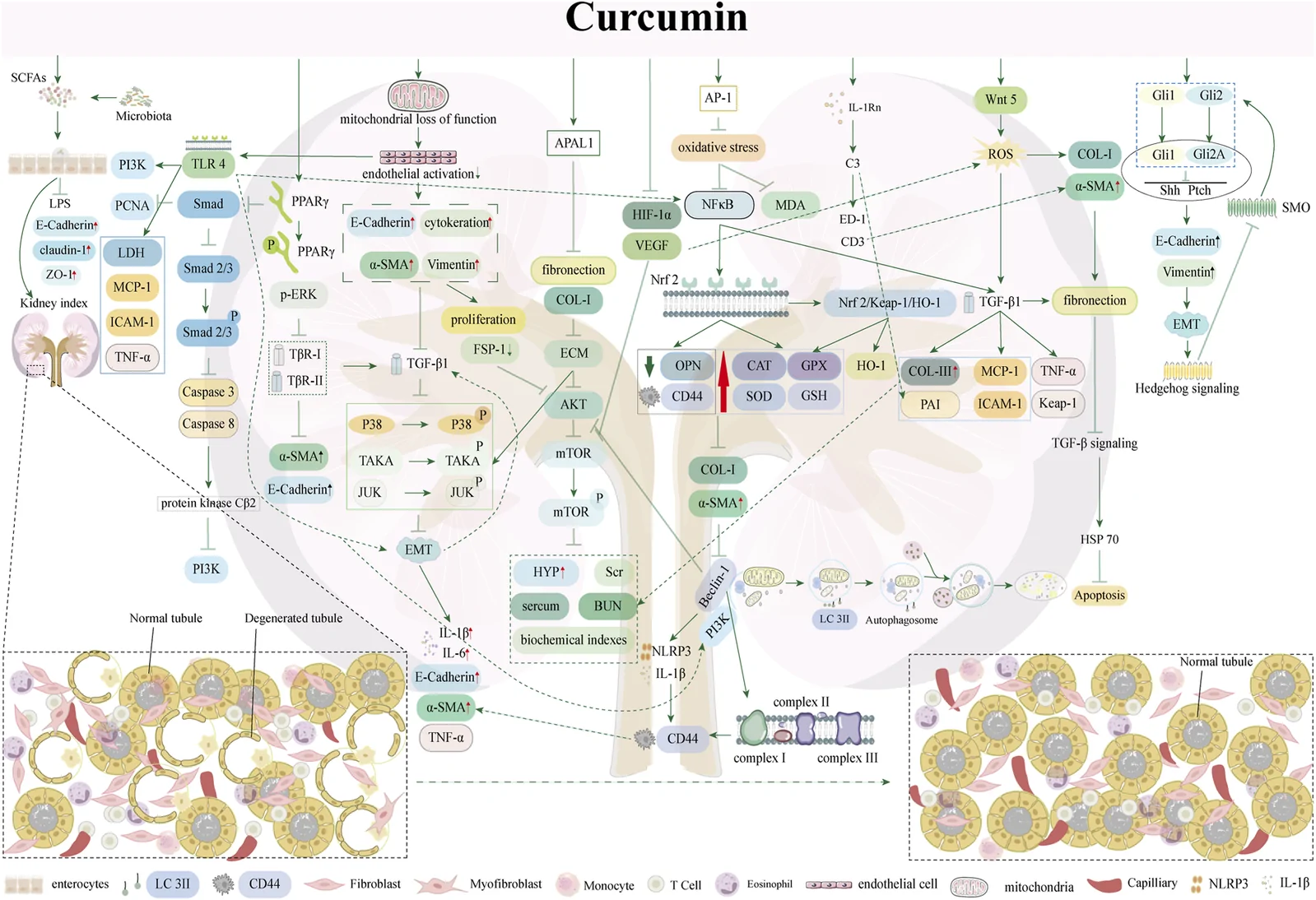
Antifibrotic role of curcumin (CUR) in urinary system fibrosis. CUR protects against tubulointerstitial fibrosis through multiple interconnected pathways: (1) inhibiting epithelial–mesenchymal transition (EMT), (2) blocking transforming growth factor β (TGF-β)/Smad and MAPK signaling, (3) activating peroxisome proliferator-activated receptor-γ (PPARγ) and suppressing inflammation, (4) improving mitochondrial function and autophagy, (5) suppressing PI3K/AKT/mTOR and hypoxia-inducible factor 1α (HIF-1α)/vascular endothelial growth factor (VEGF) axes. These coordinated effects attenuate extracellular matrix (ECM) deposition, preserve the tubular structure, and promote DKD progression.

In BALB/c mice, CUR upregulated APPL1 expression, downregulated type I collagen and fibronectin, inhibited ECM production, reduced creatinine and urea nitrogen levels, attenuated parenchymal and tubular epithelial cell injury, and mitigated fibrosis. These antifibrotic effects were confirmed *in vitro*, where CUR suppressed p-AKT, collagen I, and fibronectin in tubular epithelial cells (EpiCM-a) (Chen et al., 2018) (Figure 7; Table 3).

**TABLE 3:** Antifibrotic role of curcumin in urinary system fibrosis.

Disease	Animal/cell model	Inducer	Dosage and duration	Described effects	Pathway	Reference
Renal fibrosis	*In vitro*: HKC	TGF-β1 (5 mg/mL)	Curcumin (3.125, 6.25, 12.5, 25, and 100 μmol/L for 72 h)	↑: Retained epithelial morphology, a cobblestone growth pattern, E-cadherin, and cytokeratin ↓: Proliferation, vimentin and α-SMA, FSP 1-positive cells, p-AKT, mTOR, p70S6K, 4E-BP1, and eIF4E	AKT/mTOR pathway	Zhu et al. (2017)
Renal fibrosis	*In vivo*: BALB/c mice (n = 48)	Clamping the renal pedicles with non-traumatic microaneurysm clamps	​	↑: APPL1 ↓: Serum creatinine (SCr), urea nitrogen, tubular epithelial cell injury, kidney injury, collagen deposition, fibrotic, collagen I, fibronectin (Fn), and ECM	APPL1/AKT signaling pathway	Chen et al. (2018)
	*In vitro*: EpiCM-a	​	Curcumin (25 µM)	↑: APPL1 ↓: p-AKT, collagen І, and Fn protein	APPL1/AKT signaling pathway	
Chronic kidney disease (CKD)	*In vivo*: female C57BL/6 mice (n = 72)	Cyclosporine A (15 mg/kg, s.c.) for 14 days	Curcumin (15 mg/kg/day, i.p.) for 14 days	↓: PAI-1, α-SMA, and collagen I	-	Hu et al. (2016)
	*In vitro*: HK-2 cells	Primary	​	↓: Proliferation, PAI-1, α-SMA, and collagen I		
	*In vitro*: HK-2-Si KL (klotho) cells	Primary Si KL	​	↑: PAI-1, α-SMA, and collagen I ↓: Klotho; Dnmt 1; methylation; and CpG249, 240, and 236		
Acute kidney injury (AKI) and CKD	*In vitro*: HUVECs	TGF-β1 (5 mg/mL)	Curcumin solution (10 mg/mL) + ZnSO_4_ solution (1.61 mg/mL)	↑: HIF-1α, VEGF, ROS, and cellular survival capacity and proliferation ↓: α-SMA, collagen I, Fn, and F-actin	-	Zhang et al. (2022)
Peritoneal fibrosis	HMrSV5	Dianeal (4.25%, 200 μL)	Curcumin (20, 40, and 80 μmol/L)	↑: Cell viability and E-cadherin ↓: α-SMA, collagen I, Fn, TGF-β, p-TAK 1, p-JNK, and p-p38	EMT	Zhao et al. (2019a)
Tubulointerstitial fibrosis	*In vitro*: HK-2 cells	TGF-β1 (2.5 mg/mL)	Curcumin (10 μM)	↑: PPARγ ↓: TβR-I, TβR-II, p-ERK, p-PPARγ, α-SMA, PAI-1, and E-cadherin -: p-Smad2 and p-Smad3	ERK	Li et al. (2013)
Renal fibrosis	*In vivo*: male SD rats (n = 30 + 40)	Cisplatin (4.5 mg/kg i.p. for 21 days)	Curcumin (300 mg/kg for 21 days)	↑: Creatinine clearance, urea nitrogen, SCr, and urea levels in serum and urine, ↓: TGF-β1, α-SMA, HYP, Shh, Smo, Ptch, renal Gli1 and Gli2, fibrotic score, kidney injury score, tubular injury, and α-SMA (renal)	Hedgehog signaling	Maghmomeh et al. (2019)
Chronic renal failure	Male SD rats (n = 70)	5/6 Nephrectomy	Curcumin (75 mg/kg for 8 weeks)	↑: Body weight ↓: Biochemical indexes (24-h urine volume, SBp, 24-h urine protein, blood urea nitrogen (BUN), SCr, and serum urea), glomerular diameter, HYP, p-mTOR, mTOR, p-P70S6K1, P70S6K1, P-4E-BP1, 4E-BP1, HIF-1α, and VEGF	mTOR/HIF-1α/VEGF signaling pathway	Yangbiao He (2019)
Obstructive nephropathy	*In vivo*: male SD rats (n = 40)	Unilateral ureteral obstruction (UUO)	Curcumin (200 and 800 mg/kg for 14 days)	↑: AP-1 ↓: Renal cortical NF-κB-DNA binding activity, ED-1-positive cell, type III collagen, MCP-1, TGF-β, PAI-1, ICAM-1, RANTES, collagen III	NF-κB-dependent pathway	Kuwabara et al. (2006)
Nephrolithiasis	*In vivo*: male C57BL/6 mice (n = 32)	Glyoxylate (100 mg/kg for 1 week)	Curcumin (50 and 100 mg/kg for 14 days)	↑: SOD, CAT, GPx, GR, GSH, HO-1, NQO1, UGT, NRF2, and p62↓: Calcium oxalate (CaOx), MDA, IL-6, MCP-1, OPN, CD44, α-SMA, collagen I, collagen fibril deposition, TUNEL-positive cells, Beclin-1, LC3 II, and LC3 II/I	NRF2 signaling pathway	Li et al. (2019)
Renal fibrosis	SD rats	5/6 Nephrectomy	Curcumin (75 mg/kg for 8 weeks)	↑: Plasma creatinine, right kidney weight, CCr, NRF2, HO-1, and GPX ↓: Creatinine clearance, oxidative stress, inflammation, renal fibrosis, left kidney weight, proteinuria, BUN, Keap-1, MDA, TNF-α, COX-2, NF-κB, and TGF-β -: Body weight, total cholesterol, triglyceride, and HDL cholesterol	NRF2/Keap-1	Soetikno et al. (2012)
Renal fibrosis	*In vivo*: 20 male Wistar rats	Cisplatin (5 mg/kg) i.p.	Curcumin (200 mg/kg)	↑: CAT, NAG, GR, NRF2, claudin-2, occludin, E-cadherin, and β-catenin ↓: Plasma creatinine, BUN, MDA, NGAL, KIM-1, ATN, cleaved caspase 3, TGF-β1, collagen I, collagen IV, α-SMA, Hsp70/72, protein tyrosine nitration (3-NT), and PKC β2	Oxidative stress	Trujillo et al. (2016)
Renal interstitial fibrosis (RIF)	*In vivo*: male SD rats (n = 24)	UUO	Curcumin (200 mg/kg for 7 and 14 days)	↑: Complex I, II, III, and IV; LC3B–LC3A ratio; and Beclin-1 ↓: Tubulointerstitial injury, RIF, collagen I, collagen III, NLRP3, caspase-1, IL-1β, MAVS, VDAC1, VDAC2, autophagosomes, PI3K, AKT, and mTOR	PI3K/AKT/mTOR pathway	Lu et al. (2021a)
Chronic kidney disease (CKD)	*In vivo*: male SD rats	Monoclonal antibody OX-7 (2.2 mg/kg)	Curcumin (100 mg/kg)	↑: HO-1 ↓: PAI-1, TGF-β, Fn, proteinuria, Fn production, and glomerular PAS-positive material	TGF-β	Jens Gaedeke (2005)
AKI and CKD	*In vitro*: NRK-49F cells	TGF-β1 (5 mg/mL)	Curcumin solution (10 mg/mL) + ZnSO_4_ solution (1.61 mg/mL)	↑: ROS, cellular survival capacity, and cellular proliferation ↓: α-SMA, collagen I, Fn (Fn), and F-actin	TGF-β pathway	Zhang et al. (2022)
Renal fibrosis	*In vivo*: male C57 mice (n = 32)	UUO	Curcumin (50 and 100 mg/kg for 14 days)	↑: PPARγ ↓: Collagen fibers, Fn, collagen I, α-SMA, PCNA, and p-Smad2/3	TGF-β1 signaling pathway	Zhou et al. (2014)
	*In vitro*: NRK-49F cells	-	Curcumin (10, 20, and 30 μM)	↑: ↓: Proliferation, α-SMA, PCNA, cyclin D1, and p-Smad2/3		
	*In vitro*: NRK-52E cells		Curcumin (10, 20, and 30 μM)	Proliferation (−)		
Renal fibrosis (unilateral ureteral obstruction)	*In vivo*: male SD rats (n = 30)	UUO	Curcumin (50 and 100 mg/kg for 8 weeks)	↓: SCr, BUN levels, tubular injury, TGF-β1, P-Smad2/3, cleaved caspase-3, cleaved caspase-8, Dragon levels, and apoptosis	TGF-β1/Smad signaling pathway	Chen et al. (2021)
	*In vitro*: HK-2 cells	TGF-β1 (5 mg/mL for 48 h)	Curcumin (10, 20, and 30 μM)	↑: E-cadherin and vimentin ↓: Dragon, Fn, collagen I, collagen IV, and α-SMA		
Ischemia reperfusion injury (IRI)	*In vitro*: PC3 human prostate cells, LLC-PK1, and primary porcine kidney endothelial cells	Hypoxic atmosphere (95% N_2_, 5% O_2_, Bactal 2 gas Air Liquide)	Curcumin (65 mmol/L)	↑: Viability ↓: Mitochondrial loss of function, endothelial activation, LDH, TLR4, P-selectin, ICAM1, TNF-α, and MCP 1	TLR	Thuillier et al. (2014)
RIF	*In vivo*: male C57 BL/6 mice (n = 18)	UUO	Curcumin (50 mg/kg/day for 14 days)	↑: E-cadherin ↓: SCr, BUN, IL-1β, IL-6, TNF-α, TGF-β1, α-SMA, ECM collagen, vimentin, TLR4, and NF-κB P65	TLR4/NF-κB and PI3K/AKT signaling pathways	Wang et al. (2020)
	*In vitro*: HK-2 cells	TGF-β1 (10 ng/mL for 48 h)	Curcumin (10 ng/mL for 48 h)	↑: α-SMA and vimentin ↓: Viability, TLR4, NF-κB P65, IL-1β, IL-6, and TNF-α		
Diabetic nephropathy	*In vivo*: male Wistar rats	Streptozotocin (100 mg/kg body weight)	Curcumin (10 Mg/kg/day for 56 days)	↑: Wnt 5α, β-catenin, and TOP–FOP ratio ↓: TGF-β1 and 8-OH-d	Wnt/β-catenin/TGF-β1	Ho et al. (2016)
Uric acid nephropathy	Male Wistar rats (n = 30)	Adenine (150 mg/kg) + potassium oxonate (250 mg/kg) oral gavage for 4 weeks	Curcumin (200 mg/kg for 8 weeks)	↑: SCFAs, ZO-1, occluding, and claudin-1 ↓: Serum uric acid, SCr, BUN level, kidney index, and LPS	Intestinal flora	Xu et al. (2021)
CKD	Male SD rats (n = 48)	Adenine (0.25% w/w in feed for 35 days)	Curcumin (37.5, 75, and 150 mg/kg for 35 days)	↑: Plasma sclerostin concentrations, NRF2 (kidney), glutathione, diffuse acute tubular necrosis, interstitial fibrosis, apoptotic cells, dilatation of tubules, interstitial inflammatory cell infiltration, and tubular atrophy ↓: IL-1β, IL-6, TNF-α, and cystatin C -: BP	-	Ali et al. (2017)
IRI	*In vivo*: large white male pigs	Clamping the right renal pedicle for 60 min with a vascular non-traumatic clamp	Curcumin (12 mg/mL)	↑: IL-1Rn, complement C3, pro-fibrotic genes PAI-1, and vimentin ↓: Creatinine values (day 5, 7, 11, and 14), ED 1, CD 3, vimentin, and α-SMA	-	Thuillier et al. (2014)
AKI and CKD	*In vivo*: male SD rats	2/3 Nephrectomy	Curcumin solution (10 mg/mL) + ZnSO_4_ solution (1.61 mg/mL)	↑: vWF ↓: α-SMA, SCr, and BUN -: ALT, AST, TP, and ALB	-	Zhang et al. (2022)
CKD	*In vitro*: primary rat mesangial cells	-	Curcumin (10 μM)	↑: HO-1	-	Jens Gaedeke (2005)

#### 2.4.2 EMT

*In vitro*, human peritoneal mesothelial cells (HMrSV5) were stimulated with Dianeal (4.25%, 200 μL). CUR (20–80 μmol/L) dose-dependently enhanced cellular activity; upregulated E-cadherin; decreased α-SMA, collagen I, and fibronectin; and inhibited EMT progression. Mechanistically, CUR ameliorated renal fibrosis by decreasing TGF-β, TAK1, JNK, and p38 phosphorylation (Zhao et al., 2019a) (Figure 7; Table 3).

#### 2.4.3 ERK

Tubulointerstitial fibrosis is a major pathological feature of progressive kidney damage and a key driver of end-stage renal disease. In TGF-β1-induced HK-2 cells (2.5 mg/mL), CUR (10 μM) upregulated PPARγ; downregulated TβR-I, TβR-II, α-SMA, PAI-1, and E-cadherin; and suppressed ERK and PPARγ phosphorylation but had no effect on Smad2/3 (Li et al., 2013) (Figure 7; Table 3).

#### 2.4.4 mTOR signaling pathway

In a 5/6 nephrectomy-induced chronic renal insufficiency rat model, 8-week oral CUR (75 mg/kg) increased body weight and decreased biochemical dysfunction, glomerular diameter, and HYP content (Yangbiao He, 2019). Additionally, CUR downregulated HIF-1α, VEGF, and mTOR phosphorylation (Zhu et al., 2017). In HKC cells, CUR maintained epithelial morphology by upregulating E-cadherin and cytokeratin and downregulating vimentin and α-SMA. It also reduced cell proliferation, differentiation, and FSP-1-positive cells via suppression of the mTOR signaling pathway. In a unilateral ureteral ligation (UUO)-induced renal interstitial fibrosis (RIF) model, CUR (200 mg/kg for 7 and 14 days) enhanced mitochondrial autophagy, as evidenced by an increased ratio of complex I–IV, Beclin-1, pore-forming protein, and LC3B/LC3. This autophagic enhancement led to reduced inflammatory formation, autophagosome accumulation, aspartic protease, and IL-1β levels, thereby reducing collagen type I/III, mitochondrial antiviral signaling protein, PI3K, AKT, and mTOR expression. Consequently, CUR ameliorated tubulointerstitial injury and fibrosis. Collectively, these findings suggest that CUR coordinates the PI3K/AKT/mTOR axis with HIF-1α/VEGF and mitochondrial autophagy to counteract renal fibrosis (Lu Li et al., 2021) (Figure 7; Table 3).

#### 2.4.5 Hedgehog signaling

In SD rats with cisplatin-induced kidney injury (4.5 mg/kg), oral CUR (300 mg/kg, 21 days) improved kidney function, as evidenced by increased creatinine clearance and decreased blood creatinine, serum urea, and urine urea. CUR also decreased renal HYP levels; downregulated TGF-β1, α-SMA, renal Gli1, and Gli2; and intervened in the Shh signaling pathway. Consequently, CUR attenuated tubular and parenchymal injury and reduced renal fibrosis scores (Maghmomeh et al., 2019) (Figure 7; Table 3).

#### 2.4.6 NF-κB-dependent pathway

Obstructive nephropathy results from impaired urine flow, leading to kidney damage (Kuwabara et al., 2006). The CC chemokines RANTES and MCP-1 play a key role in this condition. In another study, obstructive nephropathy was induced *in vitro* through UUO. Oral CUR (200 and 800 mg/kg for 14 days) upregulated AP-1 expression; downregulated renal cortical NF-κB-DNA binding activity; reduced the number of ED-1-positive cells; and decreased the expression of collagen III, MCP-1, TGF-β, PAI-1, ICAM-1, and RANTES, thereby attenuating inflammation and fibrosis (Kuwabara et al., 2006) (Figure 7; Table 3).

#### 2.4.7 NRF2 signaling pathway

Nephrolithiasis is caused by the accumulation of crystalline substances in the kidney. Globally, kidney stones affect 3.5% of the population annually and 15%–25% over a lifetime. Calcium oxalate (CaOx) accounts for over 80% of cases. CaOx crystals form when glyoxylate is converted to oxalic acid, which then binds to calcium ions. These crystals adhere to renal tubular epithelial cells, disrupt cell membranes, and deposit in tubules and interstitium, thereby leading to stone formation (Li et al., 2019). To counteract this process, CUR has been investigated for its ability to mitigate CaOx adhesion and aggregation by suppressing adhesion molecule expression on tubular epithelial cells. Yinhui Li et al. reported that male C57BL/6 mice with glyoxylate-induced renal calculi received oral CUR. CUR activated the NRF2 pathway, which reduced OPN and CD44 expression, enhanced antioxidant enzyme activity, and increased HO-1 and NQO1 levels. As a result, CUR consistently exhibited kidney injury markers, fibrotic proteins, and apoptotic and autophagic signals and exerted antilithogenic effects via antioxidant, anti-apoptotic, anti-autophagic, anti-inflammatory, and antifibrotic pathways (Soetikno et al., 2012) (Figure 7; Table 3).

#### 2.4.8 TGF-β1 signaling pathway

In a rat model of anti-Thy1 nephritis (OX-7 antibody, 2.2 mg/kg), Gaedeke et al. (2005) found that CUR increased HO-1; reduced fibrin, proteinuria, and glomerular PAS-positive material; downregulated PAI-1; and inhibited TGF-β-mediated fibrosis.

In TGF-β1-stimulated HUVECs (5 mg/mL), combined with ZnSO_4_ enhanced cell survival and proliferation; upregulated HIF-1α and VEGF; reduced ROS; and downregulated α-SMA, collagen I, fibronectin (Fn), and F-actin (Zhang et al., 2022). Similar results were obtained in NRK-49F cells (Zhang et al., 2022) (Figure 5). Zhou et al. (2014) conducted *in vitro* and *in vivo* experiments on male C57 mice, inducing renal fibrosis. In a UUO-induced renal fibrosis model in male C57 mice, CUR (50/100 mg/kg for 14 days) upregulated PPARγ; decreased the accumulation of type I collagen, α-SMA, and fibrin; downregulated PCNA and p-Smad2/3; and ameliorated fibrosis by impeding local fibroblast proliferation and ECM deposition (Zhou et al., 2014) (Figure 7; Table 3). These results were validated using NRK-49F and NRK-52E cells. CUR also reduced serum creatinine (SCr) and blood urea nitrogen (BUN) levels, apoptosis, and renal tubular injury; downregulated TGF-β1, p-Smad2/3, caspase 3/8, and RGMb; and against renal fibrosis by suppressing TGF-β1/Smad signaling (Chen et al., 2021) (Figure 7; Table 3).

#### 2.4.9 TLR4/NF-κB and PI3K/AKT signaling pathways

In models of renal ischemia reperfusion injury (IRI) using PC3 human prostate cells, LLC-PK1 porcine proximal tubular epithelial cells, and porcine renal primary endothelial cells, CUR improved cell activity and mitochondrial dysfunction; attenuated endothelial activation; and decreased oxidative dehydrogenase, TLR4, P-selectin, ICAM-1, TNF-α, and MCP-1 (Thuillier et al., 2014). In a cisplatin-induced chronic kidney disease (CKD) model, CUR treatment increased plasma sclerostin and glutathione (GSH) levels, downregulated the expression of IL-1β, IL-6, TNF-α, NRF2, cystatin C, and lipocalin, attenuated oxidative stress and apoptosis, and ameliorated interstitial inflammation, tubular dilation, atrophy, and necrosis (Ali et al., 2017) (Figure 7; Table 3). In the UUO replication RIF, CUR effectively prevented EMT, ECM deposition, and inflammation through TLR4/NF-κB and PI3K/AKT signaling pathways (Wang et al., 2020). Mechanistically, CUR reduced the activity of SCr, BUN, and HK-2 cells injury; downregulated IL-1β, IL-6, TNF-α, TGF-β1, α-SMA, TLR4, NF-κB P65, and IκBα and PI3K/AKT phosphorylation; and restored E-cadherin, α-SMA, and vimentin balance (Wang et al., 2020) (Figure 7; Table 3).

#### 2.4.10 Wnt/β-catenin

In diabetic nephropathy induced by STZ, CUR attenuates diabetic glomerular damage under high glucose conditions by upregulating Wnt5α, β-catenin, and the TOP–FOP ratio, reducing superoxide synthesis, downregulating TGF-β1 and fibronectin, restoring Wnt activity, and achieving high glucose-induced TGF-β1 signal to delay ECM accumulation in diabetic glomeruli (Ho et al., 2016) (Figure 7; Table 3). In a cyclosporine A-simulated CKD, CUR reduces the expression of PAI-1, α-SMA, collagen I, and TGF-β1 in HK -2 cells and Klotho-knockdown HK-2- cells. Conversely, knockout abolished these protective effects, indicating that klotho is required for CUR’s antifibrotic action (Hu et al., 2016) (Figure 7; Table 3).

#### 2.4.11 Intestinal flora

Uric acid nephropathy is caused by the oral administration of adenine and potassium oxonate. CUR reduces renal indices, attenuates visceral histopathological changes and intestinal damage, decreases LPS secretion, ameliorates metabolic endotoxemia, and restores tight junction proteins, thereby maintaining intestinal barrier integrity. Consequently, CUR modulates the gut microbiota by increasing SCFAs, *Lactobacillus*, and *Ruminococcus* while decreasing *Lactobacillus*, *Shigella*, and *Lactobacillus acidophilus*, which collectively alleviates renal tissue damage caused by hyperuricemia (Xu et al., 2021) (Figure 7; Table 3).

#### 2.4.12 Oxidative stress

In a cisplatin-induced nephrotoxicity model, oral CUR (200 mg/kg) ameliorated kidney injury by decreasing plasma creatinine and BUN; restoring kidney function; increasing CAT, N-acetyl-beta-D-glucosidase, and GSH reductase and GR activities; upregulating NRF2 and tight junction proteins; decreasing MDA, kidney injury molecule-1, and neutrophil gelatinase-associated apolipoprotein; suppressing apoptosis and acute tubular necrosis; downregulating TGF-β1, α-SMA, collagen type I/IV, and heat shock protein 70 (HSP70), thereby delaying kidney injury progression by ameliorating oxidative damage (Trujillo et al., 2016). In a porcine renal IRI model, CUR reduced creatinine values; attenuated histological damage; improved animal survival; upregulated interleukin-1 receptor agonist (IL-1Ra), and complement C3, PAI-1; and downregulated ED1, CD3, and α-SMA (Thuillier et al., 2014). In 2/3 nephrectomy-simulated acute kidney injury to CKD, topical CUR plus zinc sulfate improved renal blood supply. Immunofluorescence revealed increased vWF and decreased α-SMA expression in the renal vasculature and tubules, suggesting neovascularization (Zhang et al., 2022) (Figure 7; Table 3).

### 2.5 Other systems

**FIGURE 8:**
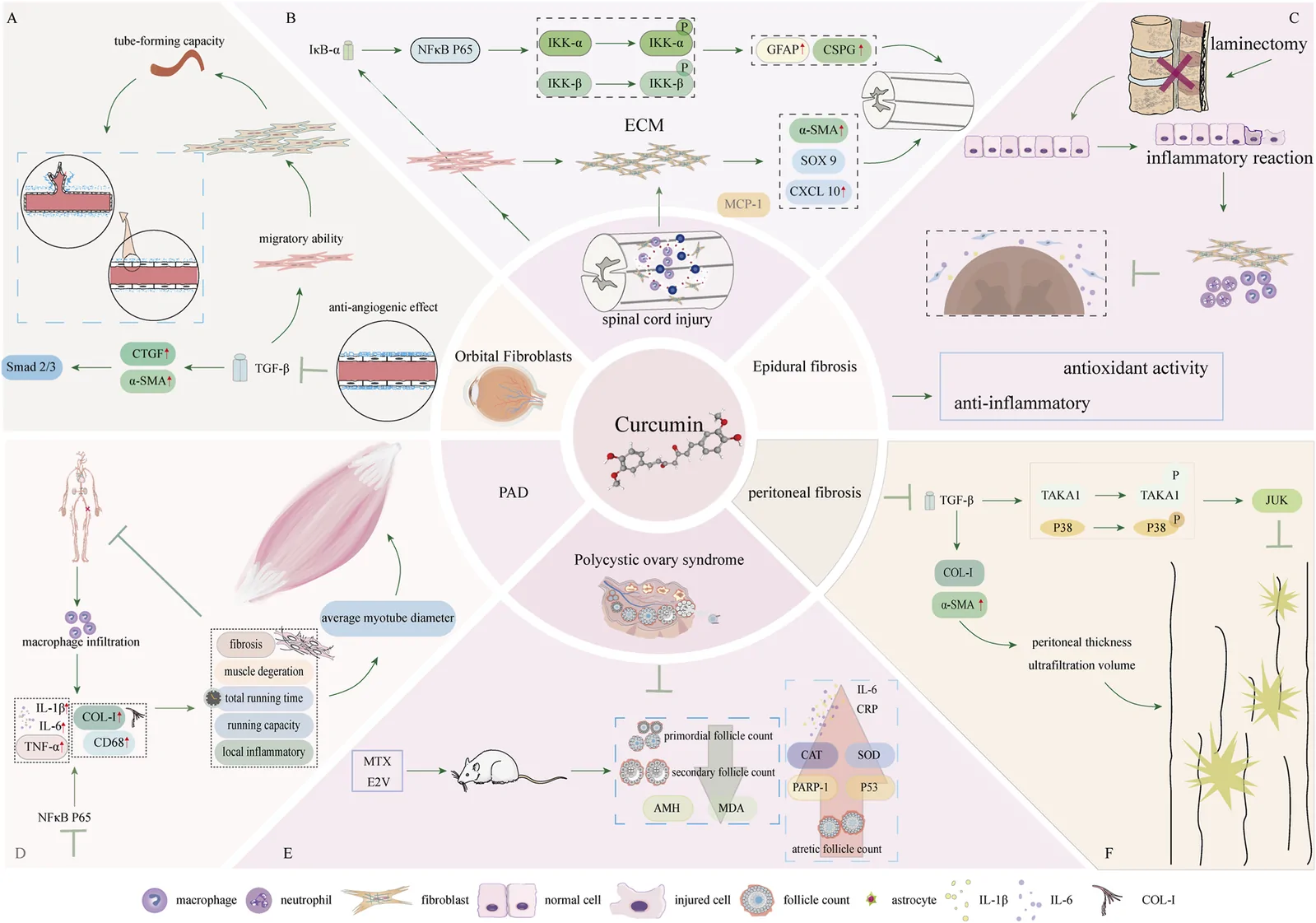
Antifibrotic role of curcumin (CUR) in other system fibrosis. CUR modulates key signaling molecules and downregulates pro-fibrotic and pro-inflammatory factors. In different pathological settings, CUR reduces macrophage/neutrophil infiltration, fibroblast activation, and fibrosis while improving functional outcomes such as tube-forming capacity, migratory ability, antiangiogenic effect, average myotube diameter, total running time, running capacity, and follicle count. **(A)** Orbital fibrosis; **(B)** Spinal cord injury; **(C)** Peripheral arterial disease; **(D)** Muscle fibrosis; **(E)** Polycystic ovary syndrome; **(F)** Peritoneal fibrosis.

In other systems, curcumin also exerts antifibrotic and anti-inflammatory effects (Figure 8). It modulates key signaling mediators, downregulates pro-fibrotic and pro-inflammatory factors, and reduces fibrosis in spinal cord injury (SCI), as well as in the peritoneum, orbit, ovary, and skeletal muscle, while improving functional outcomes such as tube-forming capacity, migratory ability, follicle count, and running capacity.

#### 2.5.1 Orbital fibrosis

In orbital fibrosis, CUR modulates the TGF-β1/Smad2/3 signaling pathway, thereby inhibiting TGF-β1-induced myofibroblast differentiation and vasoreactivity, attenuating CTGF and α-SMA expression, increasing the antiangiogenic effect, and decreasing myofibroblast differentiation and pro-angiogenic activity (Yu et al., 2021) (Figure 8A; Table 4).

**TABLE 4:** Antifibrotic role of curcumin in other system fibrosis.

Disease	Animal/cell model	Inducer	Dosage and duration	Described effects	Pathway	Reference
Peripheral arterial disease	Male C57BL/6J mice	The left femoral artery, great saphenous vein, iliac circumflex artery/vein, and muscular branch were doubly ligated	Curcumin (100 mg/kg)	↑: Running capacity and total running time ↓: Macrophage infiltration, local inflammation, fibrosis, muscle degeneration, TNF-α, IL-1β, IL-6, and NF-κB p65	NF-κB signaling pathway	Liu Y. et al. (2016)
Muscle fibrosis	Male Wistar rats	Glycerol (intramuscular injection)	Curcumin (20 mg) + polymer/sol-vent mixture	↑: Average myotube diameter ↓: Fibrosis index, CD68, and collagen I	-	Mahdy and Madkour et al. (2022)
Epidural fibrosis	Wistar albino rats (n = 32)	Laminectomy (T12 and L4 vertebral columns were removed *en bloc*)	Curcumin (100 and 200 mg/kg for 7 days)	↓: Inflammation, foreign body reaction, granulation tissue formation, medulla spinalis retraction, and epidural fibrosis formation	-	Demirel et al. (2021)
Epidural fibrosis	Female Wistar albino rats (n = 21)	Laminectomy (T9 and L3 vertebral columns were removed *en bloc*)	Curcumin (local spongostan soaked with curcumin; 100 mg/kg for 8 days)	↑: Anti-inflammatory and antioxidant effects ↓: Epidural fibrosis, macrophages, neutrophils, and fibroblasts -: Histopathological assessment and arachnoid involvement	-	Ismailoglu et al. (2018)
Orbital fibroblasts	Orbital fibroblasts	TGF-β1 (1 and 5 ng/mL)	Curcumin (0, 1, 2.5, and 5 μM)	↓: α-SMA, TGF-β, and Smad2/3	TGF-β	Yu et al. (2021)
Orbital fibroblasts	EA.hy926 endothelial cells	TGF-β1 (1 and 5 ng/mL)	Curcumin (0, 1, 2.5, and 5 μM)	↑: Antiangiogenic effect ↓: α-SMA, TGF-β, Smad2/3, and tube-forming and transwell migration capacities	TGF-β	
Orbital fibroblasts	Microvascular endothelial cell line	TGF-β1 (1 and 5 ng/mL)	Curcumin (0, 1, 2.5, and 5 μM)	↓: Migratory ability and tube-forming capacity	TGF-β	Yu et al. (2021)
Spinal cord injury	*In vivo*: female SD rats	SCI (T8–10, fixed with a 50-g aneurysm clip for 60 s)	Curcumin (100 mg/kg)	↑: IκB-α ↓: GFAP, CSPG, cystic cavity volume, astrocyte activity, NF-κB p65, p-IKK-α, p-IKK-β, and p-IκB-α	NF-κB	Yuan et al. (2017)
Spinal cord injury	*In vitro*: fastrocytes	TGF-β1 and TGF-β2 (10 ng/mL)	Curcumin (1 μM)	↓: NF-κB p65, p-IKK-α, p-IKK-β, p-IκB-α, MCP-1, RANTES, CXCL10, macrophage, T-cell infiltration, SOX 9, ECM, CSPG, and α-SMA	NF-κB	Yuan et al. (2017)
Ovarian dysfunction	Wistar albino rats	MTX (0.35 mg/kg/day)	Curcumin (200 mg/kg/day for 28 days)	↑: MDA, primordial follicle count, secondary follicle count, AMH levels, P53, and PARP-1 ↓: SOD, CAT, and atretic follicle count	-	Keçeci et al. (2025)
PCOS	Wistar rats	E2V (2 mg/kg was injected subcutaneously)	Curcumin (100 and 300 mg/kg for 14 days)	↓: Glucose, insulin, HOMA-IR, HOMA-B%, QUICKI, IL-6, CRP, collagen expression, and tissue necrosis and core	-	Mohammadi et al. (2017)

#### 2.5.2 SCI

For the *in vivo* study, SD rats were used to establish a spinal cord injury (SCI) model by clamping the T8–T10 segment with a 50-g aneurysm clip for 60 s. After curcumin intervention, IκB-α expression was upregulated; cystic cavity volume and astrocyte activity were reduced; and the expression of GFAP, CSPG, and NF-κB p65, as well as the phosphorylation of IKK-α, IKK-β, and IκB-α, were decreased. For the *in vitro* study, astrocytes were stimulated with TGF-β1 and TGF-β2; curcumin treatment attenuated macrophage and T-cell infiltration, reduced ECM deposition, and decreased MCP-1 and RANTES aggregation. (Yuan et al., 2017) (Figure 8B; Table 4).

#### 2.5.3 Epidural fibrosis

In laminectomy-replicated epidural fibrosis models in rats, CUR exerts anti-inflammatory and antifibrotic effects. It reduces inflammation, peripheral limb reaction, granulation tissue growth, foreign body reaction, and epidural fibrosis formation while ameliorating spinal cord mobility impairment (Demirel et al., 2021). Additionally, CUR decreases infiltration of macrophages, neutrophils, and fibroblasts and with no histopathological evidence of arachnoidal involvement (Ismailoglu et al., 2018) (Figure 8C; Table 4).

#### 2.5.4 Peripheral arterial disease

Peripheral arterial disease was replicated by double ligation of the left femoral artery, great saphenous artery, iliac circumflex artery/vein, and muscular branch. CUR was administered, and it increased running capacity and total running time; reduced macrophage infiltration, local inflammation, and fibrosis in the injured tissues; attenuated the degree of muscle degeneration; and reduced the expression of TNF-α, IL-1β, IL-6, and NF-κB p65 (Liu Y. et al., 2016) (Figure 8D, Table 4).

#### 2.5.5 Muscle fibrosis

In a glycerol-induced skeletal muscle fibrosis model in rats, curcumin treatment reduced the fibrosis index, CD68-positive cells, and collagen I expression, and increased the average myotube diameter (Mahdy and Madkour, 2022) (Figure 8; Table 4).

#### 2.5.6 PCOS

Shima Mohammadi (2017) administered subcutaneous injection of estradiol valerate to replicate PCOS and demonstrated that CUR serves as a protective factor in the inflammatory state associated with PCOS. CUR was observed to decrease the incidence of necrotic hepatocytes, improve insulin sensitivity, leading to a decrease in the IR index and blood glucose, and reduce liver inflammation by decreasing IL-6 and CRP levels (Shima Mohammadi, 2017). Another study was conducted in which CUR pretreated MTX-induced ovarian dysfunction, enhanced primordial and secondary follicle counts, increased MDA and AMH levels through its antioxidant properties, decreased SOD and CAT levels, reduced the atretic follicle count, and regulated the expression of p53 and PARP-1 to protect the ovarian follicle pool (Keçeci et al., 2025) (Figure 8E; Table 4).

#### 2.5.7 Peritoneal fibrosis

In a model of peritoneal fibrosis induced by intraperitoneal PD fluid, CUR (10–40 mg/kg for 4 weeks) increases the ultrafiltration volume; improves peritoneal thickness; downregulates TGF-β1, α-SMA, and collagen I; and alleviates peritoneal fibrosis by regulating the TAK1/p38/JNK signaling pathway (Zhao et al., 2019b) (Figure 8F; Table 4).

## 3 Safety and adverse effects

CUR exhibits a broad spectrum of biological properties, including anti-inflammatory, antioxidant, anti-apoptosis, antifibrosis, and anticancer, allowing it to modulate multiple signaling pathways in carious animal models of organ fibrosis (Ahmad et al., 2021). However, several fundamental limitations must be acknowledged. First, CUR has poor bioavailability, which severely limits its translational potential despite the promising findings in preclinical studies. Second, the clinical evidence remains weak, with most data derived from small sample, short-term trails lacking rigorous controls. Regarding organ safety, CUR has shown protective properties for liver and kidney functions across a wide dose range (10–400 mg/kg) in animal models (Bugyei-Twum et al., 2016; Ho et al., 2022; Kong et al., 2020; Rai et al., 2021; Sharma et al., 2022; Zhang et al., 2022). It also showed efficacy in decreasing liver enzymes and bilirubin at doses of 50–200 mg/kg in an MCD diet-induced NAFLD model (Qin et al., 2018). Similarly, CUR considerably diminished cholesterol, triglycerides, and LDL/HDL ratios in a comorbidity model of liver fibrosis and type 2 diabetes mellitus (HFD/STZ) induced by HFD and STZ (Ma et al., 2023). In an RCT of NAFLD, continuous intake of CUR (500 mg/capsule, 3 capsules once a day) for 3 months reduced patients’ waist-to-hip ratios, fibrosis score in fibroscan, FIB-4, and APRI tests (Saadati et al., 2019). Likewise, two other double-blind, placebo-controlled clinical trials indicated that daily intake of 250 or 1,500 mg CUR for 12 consecutive weeks remarkably improved liver fibrosis and steatosis and reduced blood pressure and waist circumference, serum cholesterol, glucose, and ALT (Safari et al., 2023). Dietary CUR altered the gut microbiota, decreased alpha diversity, and increased *Blautia producta* and 2-OG levels, leading to weight gain; lower body fat mass; and reductions in blood glucose, insulin, cholesterol, triglycerides, and fatty liver formation. Furthermore, a recent meta-analysis of CUR effects on metabolic dysfunction-associated fatty liver disease, based on evidence from Iran and Thailand, confirmed that CUR supplementation reduced fasting blood glucose, body mass index, and total cholesterol (Lukkunaprasit et al., 2023). CUR is a potent antioxidant compound. Its nephroprotective effects have been studied in several models of nephrochemical injury, and its effects on ALT, AST, and creatinine have been demonstrated in an OVA-induced asthma model (Islam et al., 2023; Trujillo et al., 2016). When compared with other antioxidants for the treatment of OSF degeneration, only one patient experienced facial flushing, palmar erythema, abdominal discomfort, nausea, and stomach upset (Rai et al., 2019). These adverse events were not related to the heart, liver, spleen, lungs, and kidneys, indicating a good safety profile (Liu et al., 2025; Rai et al., 2019). However, long-term safety data in humans are lacking, and most reported doses are not standardized. Future well-designed, large-scale clinical trials with rigorous endpoints are urgently needed to establish the therapeutic window and safety of CUR in fibrotic diseases.

## 4 Different dosage forms of CUR

The aqueous solubility of CUR is extremely low, contributing to poor bioavailability and instability under light, alkaline conditions, heat, and enzyme activity (Sadeghi Mohammadi et al., 2021). The retention times of CUR peaks were 6.4 and 9.6 min, and CUR emits fluorescence on excitation at 405 nm with a strong absorbance at 450 nm in the case of free CUR (Hou et al., 2024; Lee et al., 2010). Over 75% of an oral dose is directly excreted without absorption. To raise the bioavailability and therapeutic efficacy of CUR, researchers have developed different types of formulations with different materials. In the study of CEHPNPs targeting HSC, the biosafety, efficacy, and stability of CEHPNPs were excellent. In another study, the diameters in CUR-negative and CUR-positive nanomembranes were 300–320 and 600–840 nm, respectively. The CUR-negative nanomembrane was more hydrophilic than the CUR-positive nanomembrane, and both enabled MSCs to survive longer and enhanced their liver-derived differentiation (Chen et al., 2016). Additionally, in the construction of CTPP–PEG–PCL micelles targeting CUR to prolong the somatic cycling of CUR and improve its bioavailability (Zhang Pan et al., 2021). In the preparation of CUR from tristearate and polyglycolized emulsifier solid lipid nanoparticles (SLN), varying the type and concentration of the emulsifier to simulate gastrointestinal digestion found that >91% was bioavailable, and the utilization of the micelles increased by >12-fold because of the neutral surface charge that can rapidly penetrate the CUR into the epithelial cells (Ban et al., 2020). The scholars believed that the drug carrier is an inactive excipient, which may aggravate the burden of metabolism, and proposed to use phenol-rich hydroxyl-rich epigallocatechin-3-gallate (EGCG) linked by hydrogen bonding. EGCG inhibited CUR glucuronidation and improved CUR solubility; thermal, ionic, and UV stability; antioxidant activity; and oral utilization, with peak plasma and blood concentrations of 234.92 ± 14.28 ng/mL and 1362.4 ± 106.27 ng*h/mL, respectively, and targeted intestinal release to increase oral absorption (Chen et al., 2022). The bioavailable CUR content of acacia lipid-encapsulated CUR nanoparticles was 294 ± 20 μg/mL, which was much higher than that of crystals, which was 80.1 ± 2.1 μg/mL (Peng et al., 2018). The sustained release of CUR nanolipids through tauroursodeoxycholic acid (TCA)-modified nanoparticles prolonged *in vivo* circulation, and the TCA-modified nanoparticles facilitated CUR absorption via bile acid transporter protein-mediated endocytosis, which increased K_a_ by 1.09- to 1.53-fold in the small intestine, and increased duodenal, jejunal, and ileal Peff values by 1.43-, 1.71-, and 2.44-fold, respectively. Another one was the NanoCur^TM^ by Gehan Abd-Elfatah Tawfeek to avoid the suboptimal bioavailability of free CUR. These studies demonstrate that CUR can protect organ functions such as the heart, liver, and kidney and exert anti-organ fibrosis effects (Bisht et al., 2011). Despite these advances, the translational potential of different delivery systems varies considerably. Lipid-based nanocarriers, including SLNs and nanostructured lipid carriers, offer high biocompatibility and scalability, with over 10-fold increases in oral bioavailability in preclinical models, although clinical validation remains limited (Hou et al., 2024; Tian et al., 2017). Polymeric nanoparticles with active targeting ligands, such as hyaluronic acid or triphenylphosphonium (CTPP), have shown enhanced HSC targeting and therapeutic efficacy in liver fibrosis, but manufacturing complexity and long-term safety concerns may hinder scalability (Chen et al., 2022; Zhang Pan et al., 2021). Inhalable formulations, such as CURLPMPs and MOFs, have demonstrated promising local delivery for PF, achieving high lung deposition and reduced systemic side effects (Hou et al., 2024; Hu et al., 2018). This strategy is particularly attractive for respiratory diseases because of its non-invasive administration and potential for rapid clinical translation, although formulation stability and aerosol performance require further optimization. Phytochemical co-delivery systems, such as CUR combined with piperine or EGCG, offer a simpler approach by inhibiting CUR glucuronidation and enhancing intestinal absorption, with some formulations already evaluated in clinical studies for OSF (Chen et al., 2022; Rai et al., 2019). Although these co-delivery strategies may not achieve the high targeting efficiency of nanocarriers, their simplicity and favorable safety profiles make them highly amenable to clinical application. In summary, inhalable formulations represent the most promising strategy for respiratory fibrosis because of their targeted local delivery and reduced systemic exposure; ligand-modified polymeric nanoparticles offer the greatest potential for liver fibrosis by enabling active targeting to HSCs. Phytochemical co-delivery systems also provide a clinically accessible option for oral fibrotic diseases such as OSF. Future efforts should prioritize formulation scalability, long-term safety evaluation, and rigorous clinical trials to accelerate the translation of CUR-based therapies into clinical practice. Therefore, it is expected to develop novel dosage forms of CUR that avoid poor water solubility as therapeutic agents for fibrosis.

## 5 Discussion

As a medicinal and edible substance, CUR, the active metabolite of turmeric, is a polyphenolic metabolite with a multi-target and multi-pathway mechanism, making it a promising therapeutic agent for various diseases. Its favorable efficacy, high safety profile, low toxicity, cost-effectiveness, and wide availability further enhance its research value. Endowed with anti-inflammatory, antioxidant, anti-apoptotic, antifibrotic, and other biological properties, CUR shows great promise for the treatment of various diseases, particularly fibrotic disorders. Consequently, it has attracted considerable attention in modern Chinese Materia Medica pharmacology research because of its potential in combating fibrotic diseases.

In recent years, extensive studies have demonstrated that CUR exerts therapeutic effects on fibrosis in different organs, exerting antifibrotic, antioxidant, and anti-inflammatory properties through the regulation of key signaling pathways, such as TGF-β1, AMPK, IL-17, JNK, and p-ERK and DNA methylation and autophagy, and exhibits protective effects on multiple organs including the heart, liver, lungs, kidneys, and pancreas. A comprehensive review of the current literature on the antifibrotic properties of CUR indicates its potential as both a preventive and therapeutic agent for fibrotic diseases. Variations in the therapeutic outcomes of CUR at different doses for a specific fibrotic disease induced by the same agent may be attributed to the underlying mechanisms of action elucidated by researchers and the observed clinical efficacy of the drug. Although this review comprehensively summarizes the antifibrotic mechanisms of CUR, it fails to fully focus on the fundamental limitations of its clinical application, namely, low bioavailability and insufficient high-quality clinical evidence. The absence of this critical perspective may lead the review to overestimate the antifibrotic therapeutic potential of CUR, thereby compromising the objectivity of its conclusions and its clinical reference value. PF is characterized by persistent lung inflammation, alveolar structural damage, promotion of myofibroblast to fibroblast transformation, abnormal accumulation of ECM and collagen, and ultimately progression to fibrosis. Various inducers such as BLM, CP, irradiation, OVA, and SiO_2_ can trigger this pathological process through mechanisms involving apoptosis, ROS generation, TGF-β activation, and NF-κB signaling. CUR has shown considerable therapeutic potential in alleviating PF (Gouda et al., 2019; Singh et al., 2025; Smith et al., 2010) (Figure 2). Similarly, in liver fibrosis induced by CCl_4_, the intervention mechanisms of CUR have been investigated in detail, confirming its efficacy across various fibrotic stimuli. Notably, HSC activation is a pivotal event in the progression of liver fibrosis (Lian et al., 2015). Inducers like TGF-β, TAA, MCD, and CCl_4_ can promote hepatocyte fibrosis through distinct signaling pathways (Hu et al., 2020; Jin et al., 2017; Stefanska, 2012; Wang et al., 2012) (Figures 3, 4). Numerous studies have verified the efficacy of CUR in mitigating fibrosis in various organs. Taken together, CUR exhibits remarkable antifibrotic, antioxidant, and anti-inflammatory properties, highlighting its potential as a therapeutic agent for fibrotic conditions.

Remarkable progress has been made in elucidating the pathogenesis of fibrosis. However, substantial challenges remain in translating these preclinical advancements into clinical practice. (1) Current studies on the antifibrotic mechanism of CUR mainly focus on individual targets or single pathways within the same disease context, with a notable lack of in-depth exploration into the cross talk and synergistic interactions between different pathways and their corresponding molecular targets. For instance, comprehensive correlation analyses considering the shared or divergent mechanisms induced by various fibrotic stimuli are still scarce. Moreover, the molecular mechanisms of the same fibrotic disease induced by different agents remain controversial. Future investigations should aim to clarify the intricate regulatory networks formed by interacting targets under pathological conditions, which can be achieved by applying cutting-edge technologies such as single-cell spatial sequencing, transcriptomics, and proteomics across different organs and disease models. Such integrated approaches will facilitate a deeper and more systematic understanding of the multi-target and multi-pathway regulatory characteristics of CUR, laying a foundation for its further development as an antifibrotic agent. (2) The release of inflammatory mediators, elevated fibrin expression, ECM deposition, and collagen accumulation are essential phases in physiological tissue repair and fibrotic remodeling. As a key pro-fibrotic cytokine, TGF-β plays a dual regulatory role in both normal tissue homeostasis and pathological fibrotic repair. Therefore, the potential effect of long-term CUR intervention on normal physiological repair mechanisms and tissue homeostasis requires further in-depth investigation to avoid unintended adverse effects on tissue repair processes. (3) The oral bioavailability of CUR is extremely low, with approximately 75% of the oral dose being excreted without effective absorption and utilization. Consequently, there is a growing interest in developing novel CUR formulations to improve its solubility, stability, and bioavailability. This review summarizes relevant studies on capsule formulations, lipid-based carriers, transdermal patches, and nanocomposites (Zhao et al., 2019b). However, there is a noticeable gap in research on dosage forms such as microneedles, lyophilized powders, and inhalable preparations. The therapeutic efficacy of CUR in ameliorating pathological fibrosis in PF and OSF has been well-documented in preclinical and preliminary clinical studies. Considering the specific anatomical and pathological characteristics of these diseases, there is great potential for developing CUR-based nebulized inhalers for PF or topical mucosal sprays for OSF in the future. Future research efforts are expected to concentrate on the design and optimization of new nanocomposite formulations, aimed at providing high-quality preclinical and clinical evidence to support the clinical application and translation of CUR (Chauhan et al., 2016; Chen et al., 2019; Cho et al., 2013; Cui et al., 2023; Macías-Pérez et al., 2019; Tu et al., 2012; Zhang et al., 2024). (4) Numerous preclinical studies have clearly demonstrated the antifibrotic efficacy of CUR in multiple organ systems. However, the clinical studies on OSF discussed in this review were mainly limited to small sample, single-center trials involving nearly 2,000 patients with OSF (Chandrashekar et al., 2021; Deepak et al., 2021; Nerkar Rajbhoj et al., 2021; Shao et al., 2024; Yadav et al., 2014) (Figure 4). These studies lack standardized long-term follow-up data, large-scale controlled study designs, and systematic evaluations of CUR’s efficacy in fibrosis of other organ systems. Therefore, there is an urgent need to conduct large-scale multicenter, RCTs covering various organ fibrotic diseases to establish robust and reliable clinical evidence for the antifibrotic application of CUR. (5) This review systematically summarizes the mechanistic research of CUR using various animal disease models and *in vitro* primary cell culture systems. Leveraging the rapid development of technologies that can target specific disease-driving genes and molecular networks before advancing CUR to *in vivo* clinical trials. This translational approach enables an individualized evaluation of drug efficacy based on patient-specific molecular disease profiles, further promoting the development of CUR-based precision therapies for fibrotic diseases and accelerating its clinical transformation from bench to bedside.

## 6 Conclusion and prospect

In conclusion, CUR exhibits broad antifibrotic activity across multiple organs by modulating diverse signaling pathways, including TGF-β/Smad, NRF2, NF-κB, and AMPK. However, the poor bioavailability of CUR and the current lack of robust clinical evidence remain major translation hurdles. Moreover, existing clinical data are derived from small sample, short-term trials with heterogeneous dosing regimens, and long-term safety information in humans is lacking. To overcome these limitations, future research should prioritize (1) the development of novel nanocomposite or lipid-based delivery systems to enhance CUR bioavailability and target specificity, targeting key signaling pathways at both upstream and downstream levels of disease targets; (2) well-designed, large-scale, randomized controlled trials with standardized end points (e.g., fibrosis regression and event-free survival) and longer follow-up periods; (3) systematic evaluation of dose–response relationships and safety profiles in different patient populations; and (4) comparative studies across organs to identify shared versus tissue-specific antifibrotic mechanisms of CUR. Advancing the investigation of CUR and its therapeutic targets requires high-caliber basic and translational research. Furthermore, exploring novel nanocomposite formulations of CUR targeting various mechanisms and antifibrotic effects upstream and downstream of disease targets is essential. These efforts aim to provide robust evidence supporting the clinical application of CUR and facilitate therapeutic breakthroughs in the field of fibrotic diseases.
