# Supplementary material for: Antifibrotic mechanism of curcumin and its therapeutic potential in multi-organ diseases
Source: Front Pharmacol. 2026 Aug 24;17:1691999. doi: 10.3389/fphar.2026.1691999 (PMC13547310; doi:10.3389/fphar.2026.1691999)
Supplement: Supplementary file 1 [file DataSheet1.docx]

**Table S1** Antifibrotic role of curcumin in digestive fibrosis

| Disease | Animal/Cell model | Inducer | Dosage and duration | Described effects | Pathways | Refer. |
| --- | --- | --- | --- | --- | --- | --- |
| HF | HSCs | primary | CuE(0,5,10,15μM) | ↑:PPARγ,PGC-1α,p-AMPK,LXRα,SOD 2 ↓:HSC activation,PGC-1α,α1(I) collagen | AMPK pathway | (Zhai et al., 2015) |
| HF | In vivo:Male SD rats | 50% olive oil CCl4 | curcumin(100, 200, 400 mg/kg/d,for 4 weeks) | ↓:ALT,AST,ALP,LDH,HYP,HA, PC III, Collagen IV,α-SMA | AMPK/mTOR-dependent signaling pathway | (Kong et al.) |
|  | In vitro:BNL CL.2 cells | TGF-β1(2 ng/mL) | curcumin (20μM) | ↑:LC 3,Beclin-1,ATG 7,SMURF 2,AMPK,PPARγ ↓:vimentin, α-SMA ,fibronectin,TTC 3,Smads,mTOR,ROS,GSH, |  |  |
| HF | In vivo: male C57/B6 mice | TAA(100 or 200 mg/kg, three times per week,for five continuous weeks) | curcumin (20 mg/kg) | ↑:caspase-3, PARP, bax,cytochrome c ↓:AST,ALT,α-SMA,TNF-α,TIMP-1 and collagen I,bcl-2, mitochondria,PI3K,p-PI3K,Akt,p-Akt,PI3K/Akt,AMPK,p-AMPK,mTOR,p-mTOR,AMPK/mTOR,P70S6K,p-P70S6K | AMPK/mTOR-dependent signaling pathway | (Wu, Y.-L. et al., 2016) |
| HF | In vitro:HSC T6 | two-fold dilutions of CuE from 12.5 to 1.56 μM and TNF-α (10 ng/ml) for 6 or 24 h | curcumin (20μM) |  |  |  |
|  |  |  | CuE(5,10μM) |  |  |  |
| HF | In vivo:Male Sprague Dawely rats | TAA 200 mg/kg i.p. twice weekly for 18 weeks | curcumin(100,200 mg/kg) | ↑:survival percent,AFP,LC3-II,Bcl-2,Albumin concentration ↓:SOD,MDA,SQSTM 1,ALT,AST | Autophagic and Apoptic Pathways | (Elmansi et al., 2017) |
|  | In vitro:HepG2 cell | N-Acetyl Cysteine (NAC) | curcumin(50 μM ) | ↑:survival percent,AFP,LC3-II,Bcl-2,Albumin concentration ↓:SOD,MDA,SQSTM 1,ALT,AST |  |  |
| HF | Adult male Wister rats | BDL | curcumin (20 mg/kg) | ↑:Bcl 2,GSH ↓:ALT,AST,bilirubin,HYP,collagen fiber deposition,COX-2,NF-κB,MDA -:CB1,CB2 | Bcl 2 | (El Swefy et al., 2015) |
| HF | In vivo:Male balb/c mice | TAA(100 mg/kg body weight,i.p.) | curcumin (200 mg/kg) | ↑:P53,Bax,PCNA,P53 ↓: ALT,AST,serum glucose,liver damage,hepatocytes undergoing necrosis or apoptosis,collagen accumulation,TNF-α,COL1-α1,Bcl-2, -:caspase 3,TRAIL-R2 | Bcl 2 | (Stefanska, 2012) |
|  | In vitro:AML12 cells | TAA (0, 25, 50, 100, 200 and 400 mM;for 24 and 48 h) | curcumin (0, 5, 10, 20, 40 and 80 μM; for 24 and 48 h ) | ↑:P53,Bax,PCNA,P53 ↓:ALT,AST,serum glucose,liver damage,hepatocytes undergoing necrosis or apoptosis,collagen accumulation,TNF-α,COL1-α1,Bcl-2, -:caspase 3,TRAIL-R2 |  |  |
| HF | HSC-T6 | - | curcumin(20 μM ) | ↑:Fas,p53 ↓:Bcl-2 | Bcl 2 | (He et al., 2015) |
| HF | male Wistar albino rats | BPA(50 mg/kg/d,for 8 weeks) | Curcumin (100 mg/kg bw/day,p.o) | ↑:TIMP-2,CAT,GSH,IL-10,Bcl 2 ↓:ALT,AST,HYP,collagen fibers deposition,MMP-9,MDA,IL-1β,IL-1β/IL-10,caspase-3 | Bcl 2 | (Elswefy et al.) |
| HF | Male C57BL/6 mice | CCl4 (i.p. 0.1 ml/100g body weight) and olive oil (2:3 (v/v)) twice a week | curcumin (100 mg/kg) | ↑:CB 2 ↓:collagen deposition(COL-I,COL-III), pro-inflammatory cytokine expression(TGF-β,TNF-α,IL-6),liver enzyme,HYP,α-SMA,CB1 receptor,JNK, p-JNK/JNK,NF-κB p65,IκBα | CB/JNK/NF-κB pathway | (Huang et al., 2016) |
| HF | In vivo:Male balb/c mice | CCl4 (CCl4 : olive oil, 1 : 1 (V : V) at 1 mL per kg body weight ,i.p., twice weekly for 8 weeks) | curcumin(50,100,200 mg/kg) | ↓:AST,ALT,α-SMA,Col 1 α1,DNMT 1,Camk4, Fgfr3, FZD10,Gpx4, Hoxd3, Nfkb2, Prkcb, Tcfeb, | DNA methylation | (Wu, P. et al., 2016) |
|  | In vitro:HSC-T6 cell | TGF-β1(5 ng/mL) | curcumin (10,20,50μM) | ↓:α-SMA,Col 1 α1,DNMT 1,FGFR 3,FZD 10,Gpx 4,Hoxd 3 |  |  |
| HF | In vitro:HSC-T6 cell | TGF-β1(1 ng/mL) for 48 h | curcumin(20 μM ) for 48 h | ↑:PTEN,Caspase 3,miR-29 b ↓:p-ERK,p-Akt,CollA1,CollA2,Col3A1,ECM,a-SMA,DNMT 3b | DNA methylation | (Zheng et al.) |
|  | In vivo:male Sprague Dawley rats | CCl4(CCl4/olive oil, 1 : 1, v/v, per kg body weight, i.p., twice weekly) | curcumin (200 mg/kg) | ↑:PTEN,Caspase 3,miR-29 b ↓:p-ERK,p-Akt,CollA1,CollA2,Col3A1,ECM,a-SMA,DNMT 3b |  |  |
| HF | In vivo:Male Sprague-Dawley rats | CCl4 (0.1ml/100 gbodyweight) and olive oil [1:1 (w/v)] | curcumin(100,200,400mg/kg) | ↑: CBR2 ↓: CBR1,ECM(α-SMA,α(I) procollagen,fibronectin) | ECM | (Zhang et al., 2013) |
|  | In vitro:HSCs | CCl4 (0.1ml/100 gbodyweight) and olive oil [1:1 (w/v)] | curcumin (10,20,30μM) |  | ECM |  |
| HF | Male Sprague-Dawley rats | CCL4(olive oil (1:1 (v/v), 1 ml/kg body weight),for 6 weeks | curcumin (200mg/kg/day,for 6 weeks) | ↓:liver index,ALT,AST,ALP,HA,LH,PC III,α-SMA, fibronectin, α1(I) collagen | ECM/SMA | (Zhang et al., 2012) |
| HF | Adult male Wistar rats | BDL | curcumin (100 mg/kg) | ↑:IRS 1 ↓:AST, ALT, ALP,total DBIL,MDA,α-SMA, TGF-β1 ,Collagen I,IL-1β, TNF-α, iNOS ,NF-κB,FBG, insulin, HOMA-IR index,leptin,Rac 1,Rac 1-GTP,NOX 1,STAT 3,SOCS 3,ERK 1,HIF-1α,Sp1 | ERK | (Eshaghian et al., 2018) |
| HF | HSC | PDGF(20ng/ml) | curcumin (10,20,40μM) | ↓:CD31, vWF ,CD34,pro-angiogenic signal molecules (VEGF, VEGF-R2,PDGF-βR),HIF-1a,PDGF-bR,ERK,PI3K,AKT,mTOR,p70S6K,p-p70S6K,FAK,p-FAK,GTP-RhoA | ERK,mTOR,FAK/RhoA | (Zhang et al., 2014) |
| HF | Male SD rats | CCl4 (500 μL/100 g;mixed 1:1 with olive oil) body weight | curcumin (400, 1200 mg/kg),for 6 weeks | ↑:ALB,TP ↓:ALT,AST,ALP,α-SMA,Col III,HIF-1α,p-ERK | ERK/HIF-1α signaling pathway | (Zhao et al., 2014) |
| HF | In vitro: RAW264.7 cells | 400 ng/ml LPS and 2.5 ng/ml IFN-γ | curcumin (0 , 2.5 , 5, 10,20 , 40,80 μM) | ↓:CCL 7,MCP-1,CD86+,iNOS,ERK 1/2(-),p38(-),p-ERK1/2,P- p38,CD 11b + BM cells | ERK1/2 signalling | (Zhao et al., 2018) |
| Chronic cholangiopathie | Mdr2−/− mice,male | - | curcumin-enriched (4% wt/wt) diets,for 4weeks and 8 weeks | ↓:Col1α2,ALT,AP,HP,α-SMA,pERK 1/2,VCAM-1,CD 11b,CD 4+,Ki-67,K19 ↑:PPARγ -:Mcp-1,NF-κB(p65),TNF-α,CD 8+,IL-1β,TGF-β | ERK1/2 signalling | (Baghdasaryan et al.) |
| HF | male ICR mice | HFD (71% fat, 11% carbohydrates, 18% protein) | curcumin (50 mg/kg) | ↓:HIF-1α,ROS,Col1α, Col3α, fibronectin,NAFLD activity score,fibrosis,lipid deposition,β-oxidation,respiratory chain complex II,succinate accumulation,SDH activity,TGF-β1,F4/80,IL-1β,TNF-α,α-SMA | HIF-1α | (She et al., 2018) |
|  | HSCs | PA(100 μM)/dimethyl succinate for 8 h/CoCl_2_ | curcumin (10μM) |  |  |  |
| Wilson’s disease (WD) | female ATP7B knockout (Atp7b-/-) | - | curcumin(10 mg/mL) | ↑:serum cholesterol,triglyceride,HMGB 1 ↓:AST,ALT,liver and spleen indexes,hepatic inflammation and fibrosis,F4/80 +,CD 45+,p 65,CD 11b +/CD 43 +/Ly 6CHi,IL 1α,IL-1β,IL-6,IL-10,IL-12 p70,IL-23,IL-27,MCP-1,IFN-β,IFN-γ,TNFα,M-CSF | ILs | (Ho et al.) |
| Liver Pyroptosis and Fibrosis | male Jinding ducks | AFB1(60 µg/kg) | curcumin(500 mg/kg) | ↑:body weight ↓:AST,ALT,infiltrated inflammatory cells,hepatocyte disintegration,JAK 2,STAT 3,p-JAK 2/JAK 2,p-STAT 3/STAT 3,TUNEL,NLRP 3,ASC,Caspase-1,IL-1β,IL-18,α-SMA,Col-I,TGF-β | JAK 2/NLRP 3 pathway | (Cui et al., 2023) |
| HF | HSCs | leptin(100 ng/mL) | curcumin (20μM) | ↓:MAT 2A[pMAT2A (−2,954) Luc, pMAT2A (−2,847) Luc, pMAT2A (−2,752) Luc)], p-JNK,α-SMA, HSC activation,liver fibrosis | JNK pathway | (Lu, S. et al., 2021) |
|  | male C57BL/6J ob/ob mice | TAA (200 μg/g body weight, 3 times a week) | curcumin (400 mg/kg) |  |  |  |
| HF/NASH | HSC | AGEs(0,25,50,100,200μg/ml) | curcumin (0 , 5, 10,20,30μM) | ↑:AGE-R1,Leptin,Ob-R,GSH,GSH/GSSG ↓:RAGE,Nrf2,GCL,JAK 2,STAT 3,PI3K | Leptin signaling | (Tang and Chen, 2014) |
|  |  | leptin (100 ng/ml) | curcumin (0 , 5, 10,20,30μM) | ↑:AGE-R1,Leptin,Ob-R,GSH,GSH/GSSG ↓:RAGE,Nrf2,GCL,JAK 2,STAT 3,PI3K | Leptin signaling |  |
| HF | In vitro:HSCs | TAA (50 mg/kg body weight, intraperitoneally (i.p.), three times/week),for 9 weeks | curcumin(20 and 150mg/kg·wt)/CEHPNPs at a dosage of curcumin at 20 mg/kg·wt (i.p.) | ↓:MAT 2B,α-SMA,liver fibrosis,p-p38 MAPK,T-p38 MAPK,alpha1(1)collagen,HSC activation,collagen levels | MAPK pathway | (Hu and Zhou, 2020) |
|  | In vivo:C57BL/6J male mice | TAA (200 μg/g body weight,three times a week, i.p.) | curcumin (400 mg/kg,for 4 weeks) |  |  |  |
| HF | In vivo:Male SD rats | TAA(200 mg/kg,for 9 weeks) | Dehydrozingerone (DHZ),a half-structural analogue of curcuminanimals (25,50 mg/kg,for 9 weeks) | ↑:Bodyweight,total protain,survival,E-CAD, ↓:liver index,spleen index,AST,ALT,ALP,α-SMA,MMP-2,TIMP-1,COL 1α1,COL 3α1,CTGF,VIMENTIN,TIMP-3,FN1,EMMPRIN,Periostin,MPO,NE,p-P38,p-ERK | MAPK pathway | (Sharma et al.) |
|  | In vitro:HSC-LX 2 | TGF-β(5 ng/m, for 48 h) | DHZ(12.5,25,50 μM) | ↓:VIMENTIN,FN1,α-SMA,COL 1α1,TIMP-3,MMP-2,p-P38 MAPK,P38 MAPK,p-ERK,p-JNK |  |  |
| HF | adult male mice | CCL4(twice weekly for 4 weeks and then once a week for the next 4 weeks) | curcumin (5 mg/mouse/day,for 4 weeks) | ↓:miR-199 a-5p,miR-199 b*, miR-199 b,miR-200 a,miR-200 b,MMP 13,α1 procollagen | ECM | (Algandaby Mm Fau - Al-Sawahli et al.; Hassan and Al-Olayan, 2012; Wang et al., 2017) |
| HF | male SD rats | CCl4/olive oil (1:1, v/v) twice weekly at a dose of 2 ml/kg body weight | curcumin (15 mg/kg) | ↑:HGF,MMP-2,pro MMP-2 ↓:ALT,AST,AKP,T-Bil,TNF-α,IL-1b,IL-6,a-SMA |  |  |
| HF | In Vivo:male SWR mice | CCl4(1 mL/kg, 1:1 mixture with corn oil) twice weekly | curcumin (200 mg/kg) | ↑:albumin,SOD, CAT,GSH-Px,MMP-2 ↓:ALT,AST,ALP, lipid peroxidation , MDA,collagen-1, TIMP-2,TGF- 1,HYP |  |  |
| HF | In vitro:HSC-T6 cell, Alpha Mouse Liver 12 (AML- 12) | CTPP–PEG–PCL | curcumin(100 mg/mL) | ↓:MMP,collagen deposition,pseudo-lobules,hepatic lobule necrosis,cell proliferation,decreased more slowly | ECM | (Zhang, Liqiao et al., 2021) |
|  | In vivo:SD rats and Balb/c mice | CCl4(40% in olive oil, 1 mL/kg) intraperitoneally twice a week for four weeks | curcumin(17 mg/kg) | ↓:MMP,collagen deposition,pseudo-lobules,hepatic lobule necrosis,cell proliferation,decreased more slowly |  |  |
| Liver Cirrhosis | Male golden hamsters | CCl4(50 mg/kg ,two times per week during twenty weeks) | curcumin (30 mg/kg/d,for 28days) | ↑:glucose,total proteins,Nrf 2, Nrf-2/NF-κB ↓:fibrotic area, total bilirubin,AST,ALT,surface granulation,regenerative nodules,type I collagen,type III collagen,NFκB -:albumin | NF-κB | (Macías-Pérez et al., 2019) |
| HF | male ICR mice | CCl4(0.5 ml/100g) | curcumin (300 mg/kg,once per two days for 8weeks) | ↑:HMGA 1,γ-H2AX,NK P46,P 16,perforin,granzyme B,Bax,MICA, ULBP2 ↓:α-SMA,Bcl-2,f Bcl-2, Bax,  -:Cleaved-caspase 3, Cleaved-caspase 8,Cleaved-caspase 9,PVR | Bcl 2 | (Jin et al., 2017) |
| HF | Male ICR mice | CCl4 (1 mL/kg body weight) and olive oil [1:1 (v/v)] | curcumin(100, 200, 400 mg/kg/d,for 4 weeks) | ↑:PPARγ,RXRα ↓:PPARα,,α-SMA,Keap 1 | Nrf 2 pathway | (Lu et al., 2017) |
| HF | Male ICR mice | CCl4(i.p. injected every other day) | curcumin (200 mg/kg) | ↓:ALT,AST,TNF-α,IL-6,HYP,LN,HA,PC-III,IV-C |  |  |
| HF | Human LX-2 cells | Nrf 2 SiRNA | curcumin (10,20,40μM) | ↑:TG,C/EBPα, PPARγ,RXRα,RARβ,Nrf 2 ↓:PPARα,α-SMA |  |  |
| HF/Hepatic fibrosis | rats | Paraquat ,5 mg/kg | curcumin (100mg/kg) | ↑:Nrf 2, NQO1, HO-1 ↓:KEAP1, PPARγ | Nrf 2 pathway | (Kheiripour et al., 2021) |
| HF | HSCs | TAA (200 μg/g body weight,for 4weeks) | curcumin (20μM) | ↑:PPAR-γ ↓:DLK 1,Shh,α1(1) procollagen,α-SMA | PPARγ | (Qiu et al., 2014) |
| HF | In vitro:HSCs | - | curcumin (10,20,30μM) | ↑:Hhip,p-PKA,Gli1 ↓:Patched,Smo,cyclin D1, cyclin D2, and Bcl-2,CDK2,CDK 4,cyclin E1,PARP 1, caspase cascade(caspase 3,caspase 8,caspase 9,)and PARP-1,α-SMA, α1(I) procollagen, fibronectin, CTGF,C/EBPα,PPARγ,invasion and migration of HSCs,f ATP ,lactate, HK,PFK2,Glut 4,MCT 4 | PPARγ | (Lian et al.) |
| HF | male 8-week-old mice | 2 ml ­CCl4/kg b.w.),for 5weeks | curcumin (50 mg/kg) | ↑:albumin ↓:AST,ALT,PDGF,PDGFRB,TIMP-1,Acta 2,TLR-9,COL 1A 1,α-SMA | - | (Elzoheiry et al., 2022) |
| HF | male SD rats | CCL4(0.025 ml CCL4(1:6 with peanut oil)+carboxymethylcellulose sodium (10 ml/kg,5 g/l) ;thrice weekly ,for 8 weeks | curcumin(50,100,200 mg/kg) | ↓:collagenous fibers,Fibrosis score,a-SMA,apoptosis index | - | (Shu et al., 2009) |
| HF | Male SD rats | CCl4 (0.1 ml/100 g bodyweight) and olive oil [1:1 (v/v)] | curcumin(100,200,400mg/kg) | ↑:PPARγ ↓:HYP,a-SMA, a(I)procollagen, fibronectin,liver/body weight | - | (Zhang et al., 2014) |
| HF | SD male rats | CCl4 and olive oil (1:1 (v/v), 1 ml/kg body weight),three times in the fi rst week | curcumin(200 mg/kg) and/or acupuncture | ↓:hyaluronic acid(HA),laminin,procollagen III(PCIII),α-SMA,Fibronectin,α1(I) collagen | - | (Zhang et al., 2012) |
| HF | In vitro:HSC-T6 cell | - | curcumin (10μM) | ↓:α-SMA, Col1a1,Col3a1 | - | (Zhang, T. et al.) |
| Liver Cirrhosis | In vivo:Male SD rats | CCL4:2 ml of CCl4 in olive oil (1:1, v/v) per kg body weight twice weekly for up to 6 weeks | curcumin (200 mg/kg) | ↓:AST,ALT,infiltration of inflammatory,hepatic fibrosis,COL3α1, α-SMA, IL-6,TNF-α,MCP-1,HMGB 1,TLR 4,TLR 2 | - | (Tu et al., 2012) |
| HF | In vitro:HSCs |  | curcumin (5, 20, 40, 80,100 µM) | ↓:AST,ALT α-SMA, PCNA,collagen production,cell proliferation | - | (Chen et al., 2016) |
| HF | In vivo:Male balb/c mice | TAA (50 mg/kg body weight, intraperitoneally (i.p.), three times/week),for 9 weeks | curcumin(20 and 150mg/kg·wt)/CEHPNPs at a dosage of curcumin at 20 mg/kg·wt (i.p.) | ↓:AST,ALT α-SMA, PCNA,collagen production,cell proliferation | - |  |
| HF | In vivo:Male SD rats | CCl4 (0.1ml/100 gbodyweight) and olive oil [1:1 (w/v)] | curcumin(100,200,300mg/kg, for 4 weeks) | ↑:Hhip ↓:Patched,Smo,α-SMA,Shh,HSC activation,liver fibrosis | - | (Lian et al.) |
| HF | In vivo:C57/B6 male mice | carbon tetrachloride (CCl4)-injection(CCl4: olive oil=1:4, 3 μl/g CCl4 oil) twice weekly for 4 weeks | curcumin (200 mg/kg/d) | ↓:ALT,AST,collagen-I,α-SMA,TNF-α, IL-1β, IL-6,TGF-β,GRN,CCL7,MCP-1,CD11b,CD45,CD45+ leukocytes, F4/ 80+ macrophages, CD11b+ monocytes | TGF-β1 | (Zhao et al., 2018) |
| HF | Male Wistar rats | CCL4(400 mg/kg, three times a week, i.p.) for 12 weeks | curcumin (100 mg/kg body weight twice per day, p. o.) | ↓:AP,γ-GTP,FA,GSH,glycogen contents,NF-κB (p65),IL-1β,IL-10,collagen,Smad 3,α-SMA,TGF-β,CTGF,MMP-13,MMP-9,MMP-2,JNK,p-Smad 3 | Smad pathway | (Hernández-Aquino et al., 2020) |
| HCC | SD rats | DMN(10 mg/kg) | curcumin (50 or 100 mg kg) | ↑:body weight,the weight of the liver the weight of the kidney/spleen(-) ↓:GOT,GPT,f collagen I,α-SMA,MMP2,TGF-β1,p-Smad 2,p-Smad 3 -:TG或T-chol | TGF-β 1/Smad signaling | (Lee et al., 2013) |
| HF | Male SD rats | ethanol solution [30% v/v, intragastric (i.g.), 10 ml/kg/day] for 4 weeks | curcumin(200,300 mg/kg) | ↑:Smad 7 ↓:ALT,AST,ALP,collagen-I,α-SMA,fibronectin,TGF-β1,p-Smad 3 | TGF-β/Smad signaling pathway | (Chen et al., 2014) |
|  | In vitro:HSCs | - | curcumin (0, 10, 20, 30, 40, 50, 60 µM) | ↑:cell viability,GRP-78,CHOP,Smad 7 ↓:PCNA, collagen-I,α-SMA,fibronectin,p-Smad 3 |  |  |
| HF | Wistar rats | thioacetamide (TAA)(100 mg/kg) three times a week, for 8 weeks | PE (200 mg/kg) and/or curcumin (200 mg/kg) | ↓:AST, ALT, ALP,albumin,MDA,TNF-α, IL-1β, iNOS, TGF-β, MPO,α-SMA,collagen-I,NF- κB-p-65, P-Smad3,P-Smad3/Smad3 ↑:HO-1, SOD, GSH,Nrf2,Nrf2/HO-1 | TGF-β/Smad3 and NF-κB signaling pathways | (Gowifel et al., 2020) |
| HF | Male SD rats | TAA(100 mg/kg) three times a week, for 8 weeks | curcumin (200 mg/kg) | ↑:serum albumin,SOD,GSH,Nrf 2HO-1,,Nrf 2/HO-1 ↓:AST,ALT,ALP,α-SMA,collagen-1,MDA,NF-κB,p 65,TNF-α,IL-1β,iNOS,TGF-β,MPO,p-Smad 3 |  |  |
| HF | hBM-MSCs | MSC | cur+ nanoscaffold | ↑:HGF,EGF,VEGF,AFP,ALB,CK 18,HNF 4 α,α-1AT,CYP 1A 1,HGF, transferrin, fibronectin,urea,MMP-9,IL-10,SOD1 ↓:TGFβ1,FGF-2,α-SMA,TNF-α,IL-6 | TGF-β1 | (Tawfeek et al.) |
| HF | adult male Wistar rats | BDL | curcumin (100mg/kg/day,for 4weeks) | ↑:miR-21 ↓:TGF β1,miR-122 | TGF-β1 | (Nozari et al., 2020) |
| HF | Male SD rats | CCL4 (40% in olive oil) at a dose of 0.75ml/kg,for 7 weeks | - | ↑:IL-10 ↓:collagen fibers,NF-κB,TNF-α,IL-1β,IL-6,TGF-β1,HYP | TGF-β1 | (Shu-Ju Wu, 2010) |
| Liver Cirrhosis | male Rattus rattus L. rats | CCl4 (3 ml/kg, two times weekly,for 6 weeks) | curcumin(150,200,250mg/kg,for 4 weeks) | ↑:IL-10,immune system ↓:AST,ALT,TNF-α,TGF β1,GOT,GPT | TGF-β1 signaling pathway | (Abo‐Zaid et al., 2020) |
| HF | In vivo:Male SD rats | CCl4 :2 ml of CCl4/olive oil (1:1, v/v)/kg body weight ,twice/week for 6 weeks | curcumin (200 mg/kg) | ↑:Smad 7, Smad2, Smad3, ↓:inflammation, necrosis, fibrosis, micronodular fibrosis, architectural distortion, type I collagen, α-SMA, TGF-β1, p-Smad2, CTGF | TGF-β1 signaling pathway | (Qun-yan Yao, 2012) |
| HF | Male Wistar rats | CCl4 (0.1 ml/100 g body weight) and olive oil (1:1, v/v) twice/week for 8 weeks | curcumin (200 mg/kg) | ↑:MMP-13, GSH, glutathione ↓:ALT,AST,GCT,bilirubin,gamma glutamyl transpeptidase, TGF-α,collagen deposition, MDA | TGF-β1 signaling pathway | (Morsy et al., 2011) |
| Liver Fibrosis and Type 2 Diabetes | Male SD rats | a high-fat diet+streptozotocin injection(30 mg/kg dissolved in citrate buffer) | L6H4, a curcumin analogue(0.2 mg/kg/day for 8 weeks) | ↑:weight,Albumin,MMP-2 ↓:cholesterol, triglycerides,LDL/HDL ratio,ALT,AST,inflammatory cell infiltration,adipose droplets,collagen fibers,TGF-β1,TIMP-2 | TGF-β1 signaling pathway | (Ma et al.) |
| HF | In vitro:AML12 cells | TAA (0, 25, 50, 100, 200 and 400 mM) for 24 and 48 h | curcumin (0, 5, 10, 20, 40 and 80 μM) | ↑:TUNEL-positive cell,P 53,apoptotic cells ↓:HSC activation,COL1-α1,α-SMA,PCNA,Bcl 2, -:caspase 3,TRAIL-R2,necrotic cells | TIMP | (Wang et al.) |
| NAFLD | Male C57BL/6 mice | methionine and choline (MCD diet) | curcumin (0.1 mg/ml,25mg) | ↑:body weight ↓:ALT,MCP-1,CD 11b,type I procollagen,TIMP-1,α-SMA,ROS | TIMP | (Vizzutti et al., 2010) |
| HF | Male SD rats | 2 ml of CCl4 in olive oil (1:1, v/v)/kg body weight twice weekly for up to 6 weeks | curcumin (200 mg/kg) | ↓:AST,ALT,collagen-I,collagen-III,α-SMA,vWF,CD 31,VEGFR-2,collagen-IV, laminin, fibronectin,HIF-1 α,HIF-1 β,VEGF,VEGFR-1,COX 2,PlGF | VEGF | (Yao et al.) |
| HF | In vitro:HSCs | - | curcumin(20 μM ) | ↓:β-catenin protein,pGL3-OT/pGL3-OF,DLK 1 | Wnt/β-catenin pathway | (Cui et al., 2014) |
| HF | In vivo:Male SD rats | TAA (200 μg/g body weight) | Curcumin (400 mg/kg body weight) | ↓:β-catenin protein | Wnt/β-catenin pathway |  |
| HF | HSCs | 0.5mg/mL 3-(4,5-dimethylthiazol-2-yl)-2,5-diphenyl tetrazolium bromide | curcumin(20 μM ) | ↑:PPARγ, C/EBPα,RXRα, RARβ ↓:Frizzled, β-catenin | Wnt/β-catenin pathway | (Zhang et al., 2016) |
| HF | Male Wistar rats | BDL | curcumin (200 mg/kg),for 3 weeks | ↓:AST,ALP,urea,Total bilirubin,conjugated bilirubin,NF-κB(p65),iNOS -:creatinine | - | (Barta et al., 2015) |
| HF | In vitro:kupffer cells (KCs) | 400 ng/ml LPS and 2.5 ng/ml IFN-γ | curcumin (0 μM, 2.5 μM, 5 μM, 10 μM) | ↓:CD86+,iNOS | - | (Zhao et al., 2018) |
| HF | 40 female rats | ABEM(26 mg/kg/d,for 28days) | curcumin (30 mg/kg/d,for 28days) | ↑:body weight ↓:AST,LDH,HDL,LDL,triglyceride,total cholesterol,inflammation,liver fibrosis,caspase-3,Bax,TNF-α | - | (Huyut et al., 2022) |
| HF | NIH/3T3 cells | 2 mL of 10mM NaOH | curcumin(2,1.5,1,0.75,0.5,0.25mM) | ↓:collagen protein | - | (Yu et al., 2019) |
| HF | In vivo:Male SD rats | CCL4:(1.5 ml of CCl4 in olive oil (2:3,v / v) per kg body weight twice weekly)for 6 weeks | curcumin(50,100,200 mg/kg) | ↓:ALT,AST,TBIL,HA,LN,PC III,CXCL12,CXCR4,α-SMA,RhoA | - | (Qin et al., 2018) |
|  | In vitro:HSC-T6 cell | 10 ng/ml PDGF-BB | curcumin (20,40μM) | ↓:α-SMA,RhoA,COL I,CXC12/CXCR4,HSC cells migration and proliferation |  |  |
| HF | In vivo:Male balb/c mice | TAA, 100 mg/kg body weight by intrperitoneal injection | curcumin (300 mg/kg),for 8 weeks | ↑:P 53,Bax, ↓:ALT,AST,TNF-α,COL1-α1,Bcl 2,liver-to-body weight ratio,glucose -:BUN | - | (Wang et al.) |
| HF | Albino female rats | CCl4(1 ml/kg diluted by olive oil 1:1 twice a week,for 8 weeks) | hBM-MSCs(10 ×10^6^ P3 cells/rat) | ↑:ALB ↓:AST,ALT,total bilirubin,collagen I | - | (Tawfeek et al.) |
| T2DM | - | - | - | ↑:PPARγ, GCL,GSH ↓:glucose tolerance, insulin resistance, hyperglycaemia- and hypercholesterolaemia-associated liver fibrosis,NF-kB,WNT | - | (Stefanska, 2012) |
| MAFLD | Systematic reviews and meta-analyses (SRMA) and randomized controlled trials (RCT) | - | - | ↓:FBS(fasting blood sugar), body mass index, total cholesterol,AST,ALT,hepatic steatosis,BMI,TC -:HbAlc,LDL,DL,TG,BP(SBP,DBP) | - | (Lukkunaprasit et al., 2023) |
| NAFLD | ﬁfty two patients with NAFLD | - | curcumin(1500 mg,for 12 weeks) | ↑:physical activity ↓:weight, BMI, waist circumference,hip circumference, energy intake,Hepatic ﬁbrosis, serum cholesterol, glucose ,ALT -:serum insulin concentration, insulin resistance | - | (Saadati et al., 2019) |
| ALD | In vivo:C57/B6 male mice | 5% ethanol,i.v. five times a week for 2 weeks | curcumin (1 mg/ml,Curcumin powder 36.8 mg (Sigma-Aldrich, USA) was dissolved in 1 mL sodium hydroxide solution 0.5M) | ↓: ALT,AST ,TNF-α,TGF-β,NF-κB | NF-κB | (Zhao et al., 2012) |
|  | In vitro:HSC T6 | TGF-β1(1 ng/mL) for 48 h | curcumin (0, 10, 20, 30, 40 µM) | ↓: ALT,AST ,TNF-α,TGF-β,NF-κB |  |  |
| Schistosomiasis | BMSCs | infected with 90 cercariae of S. mansoni | curcumin(80 mg/ml) | ↓:Granuloma size Area,Area percentage of collagen fibers,Area percentage of OV-6 | - | (Alsulami and Abdel-Gaber, 2020) |
| HF | Male Swiss albino mice | S. mansoni- induced liver ﬁbrosis | curcumin (300 mg/kg) | ↓:ALT, AST, bilirubin,albumin,HYP | - | (El-Agamy et al., 2011) |
| NAFLD |  | patients into intervention (n=25) or control(n=21) groups | - | ↓:fibrosis score in fibroscan,FIB-4,APRI tests,BMI,Energy intake | - | (Saeede Saadati 2018) |
| NAFLD | participants | The inclusion criteria were the willingness to participate in the study; age between 18 and 65 years; and non-alcoholic fatty liver disease (grades 1–3) diagnosed by previous ultrasound. | Curcumin, at a dose of 250mg per day,for 12 weeks | ↓:waist circumference(WC),blood pressure(BP), steatosis , fibrosis ,steatosis -:weight,BMI,liver enzymes and lipid profiles | - | (Safari et al., 2023) |
| PCOS | Wistar rats | E2V(2 mg/kg was injected subcutaneously ) | curcumin (100,300mg/kg for 14days) | ↓:Glucose,Insulin,HOMA-IR,HOMA-B%,QUICKI,IL-6,CRP,collagen expression,tissue necrosis and core | - | (Shima Mohammadi, 2017) |
| Cirrhosis | In vivo:Male C57BL/6 mice | CCL4(1 µg/g of body weight,CCl4 :olive oil = 1:9,intraperitoneal injection) | curcumin(25 mg +200 µl PBS) | ↑:PPAR-γ ↓:IL-6,TNF-α,HYP,oxidized GSSG,Type I α collagen,fibronectin 1,TGF-β,cleaved-caspase 3,hepatic stellate cells -:GSH | PPARγ | (Bisht et al., 2011) |
| hepatobiliary fibrosis | old male Syrian golden hamsters | 50 metacercariae of O. viverrini(oral inoculation) | Curcumin(1% curcumin+corn-oil ,w/w) | ↑:MMP-13, MMP-7, IL-1β ↓:the dilatationofbile ducts,the thickness ofperiductal fibrosis,α-SMA,HYP,collagen I,collagen III,TIMP-1, TIMP-2, TNF-α -:MMP-2,MMP-9 | - | (Pinlaor et al., 2010) |
| PDF | Male golden hamsters | 50 metacercariae of O. viverrini(oral inoculation,for 1 month) | curcumin (50 mg/kg) | ↑:CD 10,fibronectin,abcc 2,abcb 11,abcc 11,cy7a1 ↓:HYP,α-SMA, ALT,MMP-9MMP-13,CTGF, collagen I and III, TGF-β,TNF-α,TIMP-1, 2, 3,shp-1 -:MMP-2,MMP-7 | - | (Charoensuk et al., 2016) |
|  |  |  |  | ↑:MMP-7,MMP-13,CD 10,abcc 2,abcb 11,abcc 11,cy7a1 ↓:HYP,α-SMA,ALT,MMP-9, CTGF,fibronectin, collagen I and III, TGF-β,TNF-α,TIMP-1, 2, 3,shp-1 -:MMP-2 |  |  |
|  | peribiliary myofibroblast cells | Post-infected cells from animal experiments | curcumin(10,20,40μM) | ↓:α-SMA, fibronectin,CTGF,vimentin |  |  |
| Intestinal fibrotic stricture | SD rats | 5% TNBS(10mg, 15 mg, 20mg,25mg, and 30mg ,on the 1st, 8th, 15th, 22th, and 29th day) | curcumin (50,100,200mg/kg,for 35 days) | ↑: E-cadherin,PPARγ ↓:fibrosis,inflammatory cells infiltration,collagen deposition,α-SMA,TGF-β, fibronectin,CTGF | EMT | (Xu et al., 2017) |
| Intestinal fibrotic stricture | IEC-6 cell line | TGF-β1(10 ng/mL) | curcumin(2.5,5,10μM) | ↑: E-cadherin,PPARγ ↓:α-SMA, p-Smad 3 | EMT |  |
| Peritoneal fibrosis | Male SD rats(n=70) | dibutyltin dichloride (DBTC) solution(8 mg/kg body weight,caudal vein) | curcumin (200 mg/kg/day,for 2 or 4 weeks) | ↓:interstitial fibrosis,α-SMA | - | (Lu et al.) |
|  |  | 4.25% glucose-based standard PD fluid(100mL/kg,for 8 consecutive weeks,i.p.) | curcumin (10,20,40 mg/kg,for 4 weeks) | ↑:ultrafiltration volume (UFV) ↓:MTG,peritoneal thickness,TGF-β1, α-SMA ,collagen I,TAK1, JNK,p38,p-TAK1, p-JNK,p-p38 | TAK1/p38/JNK pathway | (Zhao et al., 2019b) |
| OSMF | 40 patients clinically diagnosed with OSMF | - | 2% curcumin gel | ↑:mouth opening,tongue protrusion,cheek flexi-bility, ↓:burning sensation,LDH | - | (Chandrashekar et al., 2021) |
| OSMF | 6 clinical trials,298 patients | - | Curcumin(250,300,400mg) | ↑:mouth opening, ↓:burning sensation,epithelial hyperplasia, inflammatory cells, hyalinization of connective tis- sue, mitotic figures | - | (Al-Maweri, 2019) |
| OSMF | 40 patients clinically diagnosed with OSMF | - | Curcumin tablets (300 mg,for 3 months) | ↑:mouth opening,interincisal distance, ↓:burning sensation | - | (Yadav et al., 2014) |
| OSMF | 30 patients clinically diagnosed with OSMF | - | curcumin(Longvida lozenges;400 mg lozenges,given for 3 months duration; follow‑up for 6 months) | ↑:↑:mouth opening,interincisal distance ↓:VAS scale with spicy and normal food | - | (Hazarey et al., 2015) |
| OSMF | 119 patients clinically diagnosed with OSMF | - | curcumin(300 mg and piperine 5 mg;in both systemic or (and) topical forms;for 12 weeks | ↑:mouth opening,tongue protrusion (TP) ↓:burning sensation, | antioxidants | (Rai et al., 2019) |
| OSMF | 40 patients clinically and histopathologically diagnosed with OSMF | - | BIOCUMIN (Biochem India) twice a day for 3 months;curcumin 500 mg, piperine 5 mg, lycopene 25 mg | ↑:mouth opening,tongue protrusion (TP),mucosal flexibility (MF),reepithelialization,number of blood vessels in the connective tissue ↓:burning sensation,Col1A1,hyalinization | - | (Mahato et al., 2019) |
| OSMF | 60 patients clinically diagnosed with OSMF | - | Curcumin tablet (300mg) twice daily,given for 6 months, follow‑up for 6 months | ↑:mouth opening,tongue protrusion (TP),cheek flexibility ↓:burning sensation | - | (Piyush et al., 2018) |
| OSMF | Of these 11 studies, involving 428 patients, 7 were randomized control trials (RCTs), 1 was a nonrandomized trial, and 3 were observational studies. | - | Turmeric(C. longa 400 mg tablets,capsules of 400 mg turmeric with 100 mg black pepper,curcumin 1 g caplets,capsules of turmeric oil, turmeric oleoresin, and turmeric extract) | ↑:mouth opening,tongue protrusion,serum superoxide dismutase,Vitamins C and E ↓:burning sensation,alondialdehyde (MDA), 8‑hydroxydeoxyguanosine (8‑HOdG) -:liver function and kidney function | - | (Rai et al., 2021) |
| OSMF | 90 patients clinically diagnosed with OSMF(Six studies :2 of OL and 4 of OSF) | oral leukoplakia (OL) ;oral submucous fibrosis (OSF) | Curcumin(3.6gram/day,over 3 months,given for 6 months,follow‑up for 9 months) | ↑:mouth opening,cheek flexibility ↓:burning sensation | - | (Ahmad et al., 2021) |
| OSMF | 147 patients clinically diagnosed with OSMF | - | ①Capsule S M Fibro(twice daily for 12 weeks); ②Turmix Tablet(300 milligrams of curcumin and 5 mg of piperine) 3 times per day for 12 weeks; ③Turmix Mouth Wash (Sanat Products) 2 times per day for 12 weeks | ↑:mouth opening,tongue protrusion ↓:burning sensation | - | (Brignardello-Petersen, 2020) |
| OSMF | 60 patients clinically diagnosed with OSMF | - | Curenext oral gel;3-4 times a day,for 6 months | ↑:mouth opening ↓:burning sensation -:Stages of OSMF | - | (Nerkar Rajbhoj et al., 2021) |
| OSMF | 685 patients:male (575,83.9%) ,female(110 ,16.1%) | 13 RCTs | Curcumin | ↑:mouth opening,tongue protrusion ↓:burning sensation | - | (Shao et al.) |
| OSMF | 34 patients clinically diagnosed with OSMF | - | Curcumin (2gm/day,for 12 weeks) | ↑:mouth opening,cheek flexibility,tongue protrusion | - | (Adhikari et al., 2022) |
| OSMF | 30 patients clinically diagnosed with OSMF | - | Curcumin tablet (500 mg;given for 3 months,follow‑up for 6 months) | ↑:mouth opening ↓:burning sensation | - | (Deepak et al., 2021) |
| OSMF | 28 patients clinically diagnosed with OSMF:male (23) ,female(5) | - | curcumin and piperine 300 mg (Turmix; Sanat Pharmaceuticals; Bulandsheher, Uttar Pradesh, India) twice daily for a period of 9 months | ↓:p53, TGF-­β, iNOS | - | (Gupta et al., 2016) |
| OSMF | In vitro:normal oral mucosal fibroblasts | arecoline(20 mg/ml) | curcumin (5, 10, 20, 30 μM) for 48 h | ↑:MMP 2,Bax,apoptosis ↓:HIF-1α,TGF-β,CTGF,Bcl-2,collagen type I alpha 1,collagen type III alpha 1, tissue inhibitor of metalloprotease 2,cell proliferation and migration | HIF-1α/TGF-β/CTGF axis | (Zhang, Lin et al., 2021) |
